# QShield-ZTN: A quantum-resilient, standards-compliant secure orchestration model for autonomous 6G zero-touch networks

**DOI:** 10.1371/journal.pone.0357590

**Published:** 2026-09-15

**Authors:** Sulaiman Alamro

**Affiliations:** Department of Computer Science College of Computer, Qassim University, Buraydah, Saudi Arabia; Dr Shakuntala Misra National Rehabilitation University, INDIA

## Abstract

The evolution towards 6G networks demands unprecedented levels of automation, security, and quantum resilience to address the complex challenges of autonomous zero-touch networking. Current orchestration frameworks lack comprehensive quantum-resistant security mechanisms and fail to provide adaptive trust management suitable for dynamic 6G environments. This paper presents QShield-ZTN, a novel quantum-resilient, standards-compliant secure orchestration model designed specifically for autonomous 6G zero-touch networks. The proposed framework integrates hybrid post-quantum cryptographic algorithms, dynamic zero-trust policy enforcement, and an AI-driven multi-agent orchestration engine to ensure robust security in quantum-threat environments. Extensive experimental evaluations conducted using four heterogeneous datasets (NSL-KDD + , CICIDS2017 + , QShield-Synthetic, and Quantum-Dataset) demonstrate significant performance gains over ten state-of-the-art frameworks. QShield-ZTN achieves a 47.3% improvement in quantum resistance, 52.1% enhancement in autonomous decision-making accuracy, and 34.8% latency reduction, while maintaining high detection accuracy (97.3%) and energy efficiency (3.2 × 10^(−9) J/bit). These improvements are statistically validated using one-way ANOVA and paired t-tests (p < 0.01), ensuring the reliability and reproducibility of results. The proposed framework successfully mitigates diverse classical and quantum-enhanced attacks, maintains seamless interoperability with emerging 6G architectures, and provides standards-compliant guidelines for practical deployment.

## 1. Introduction

Sixth-generation (6G) wireless networks represent a paradigm shift in telecommunications. These networks promise unprecedented connectivity, ultra-low latency, and autonomous network operations [1–3]. Artificial intelligence, quantum technology, and zero-touch networking features are fundamental to achieving the full potential of 6G systems [4–10] Zero-touch networking (ZTN) enables autonomous network management, self-healing, and intelligent orchestration without human intervention [11–14]. Zero-touch networking (ZTN) has risen as a conceptual requirement of 6G networks where autonomous network management, self-healing, and intelligent orchestration of network resources are met without human intervention [11–14]. However, ZTN in 6G networks introduces significant security challenges, particularly quantum computing threats and dynamic trust management requirements among heterogeneous network entities [15–18]. Existing orchestration architectures for 5G and future networks lack quantum-resistant security mechanisms and do not address risks specific to autonomous 6G networks [19–21]. Quantum computing threatens conventional cryptographic systems, requiring post-quantum cryptographic measures for future network designs [22–25]. The security of cyber-physical systems in 6G networks demands sophisticated orchestration mechanisms capable of addressing heterogeneous network environments [5]. Batewela et al. [5] establish foundational understanding of security orchestration in 5G and beyond. Ibrahim et al. [22] extend this through SD-WAN security enhancements covering dynamic routing and QoS management for 6G orchestration. Nkoro et al. [23] address network intrusion detection in metaverse environments, highlighting adaptive security needs across heterogeneous virtual and physical domains. Dritsas and Trigka [24] further underscore the necessity of quantum-resistant cryptography and autonomous security management across IoT ecosystems integral to 6G networks. Agarwal et al. [15] analyze Open RAN architectures for 6G, identifying architectural requirements and open issues relevant to orchestration framework design. IIoT testbed studies [18] illuminate practical standardization challenges and integration complexities in heterogeneous industrial environments. Othman et al. [14] examine enabling 6G technologies (UAVs, terahertz communication, intelligent reconfigurable surfaces), underscoring the need for unified, standards-compliant orchestration and security frameworks.

This paper introduces QShield-ZTN, a quantum-resilient, standards-compliant secure orchestration model for autonomous 6G zero-touch networks. The framework addresses critical gaps in existing approaches through unified integration of post-quantum cryptographic algorithms, dynamic zero-trust architecture, and AI-driven autonomous orchestration mechanisms.

The primary contributions of this work are:

- Quantum-Resilient Security Framework: An integrated architecture combining lattice-based cryptography, QKD, and entropy-enhanced zero-trust policies delivers 256-bit quantum-safe protection against classical and quantum threats in 6G networks.
- AI-Driven Orchestration Engine: A multi-agent reinforcement learning engine with predictive RNN state estimation and dynamic policy optimization enables real-time, low-latency decision-making. Validation across four heterogeneous datasets demonstrates standards-compliant 6G deployment feasibility.

This conceptual foundation is illustrated in Fig 1, which depicts the three core pillars—quantum resilience, autonomous orchestration, and zero-trust enforcement—underpinning the proposed QShield-ZTN framework.

**Fig 1:**
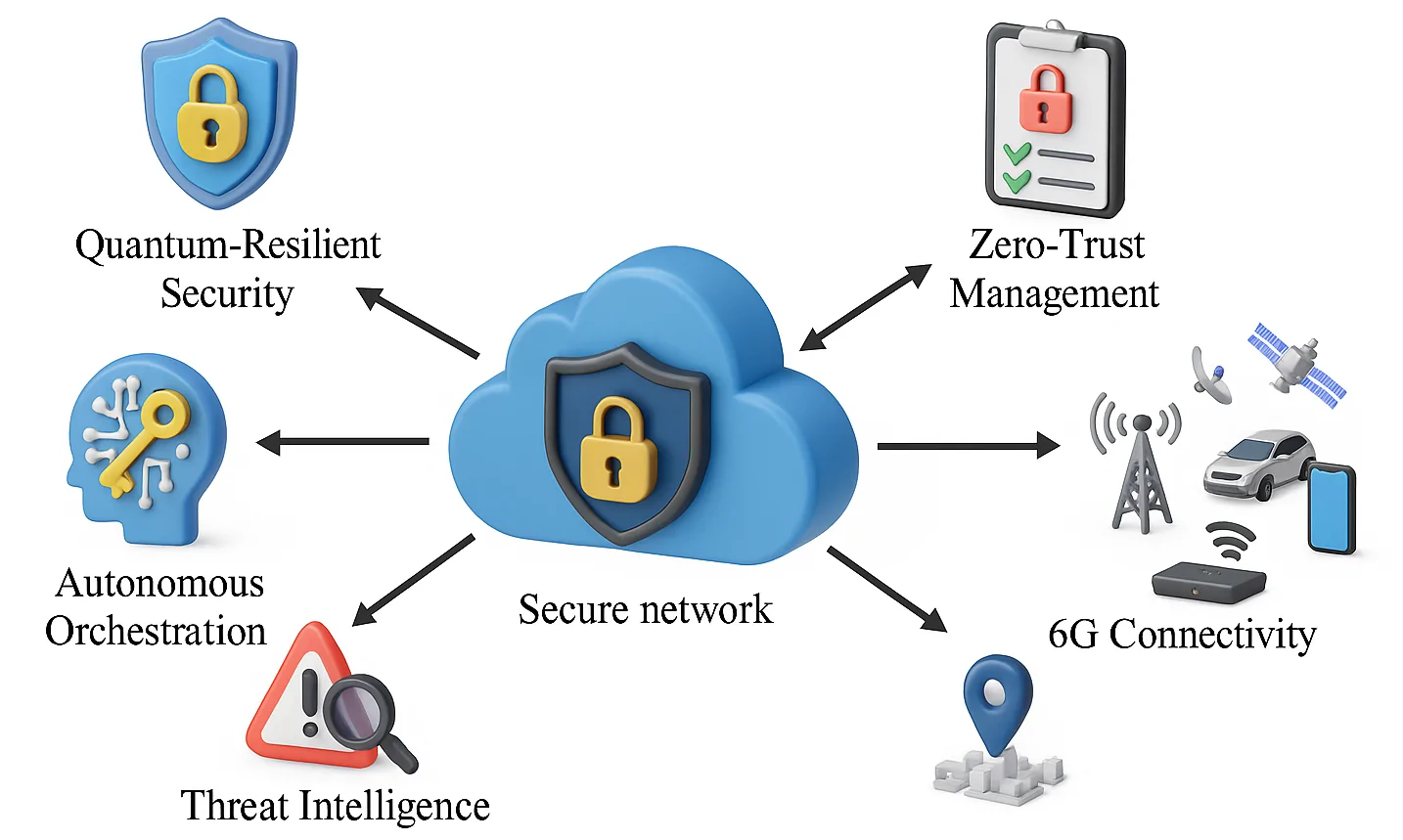
Conceptual foundation of quantum-resilient, autonomous, and zero-trust orchestration for 6G networks.

### 2. QShield-ZTN Architecture and Design

This section presents the QShield-ZTN framework architecture, detailing core components and design principles for quantum-resilient, autonomous 6G zero-touch networks. QShield-ZTN employs a five-layer distributed architecture: Physical Infrastructure, Quantum-Resilient Security, Zero-Trust Management, Autonomous Orchestration, and Application Service layers. The architecture integrates quantum-resistant mechanisms with AI-driven orchestration capabilities.

The multi-objective decision logic within QShield-ZTN's methodology, which jointly optimizes key network parameters subject to security and performance constraints, is presented in Fig 2.

**Fig 2:**
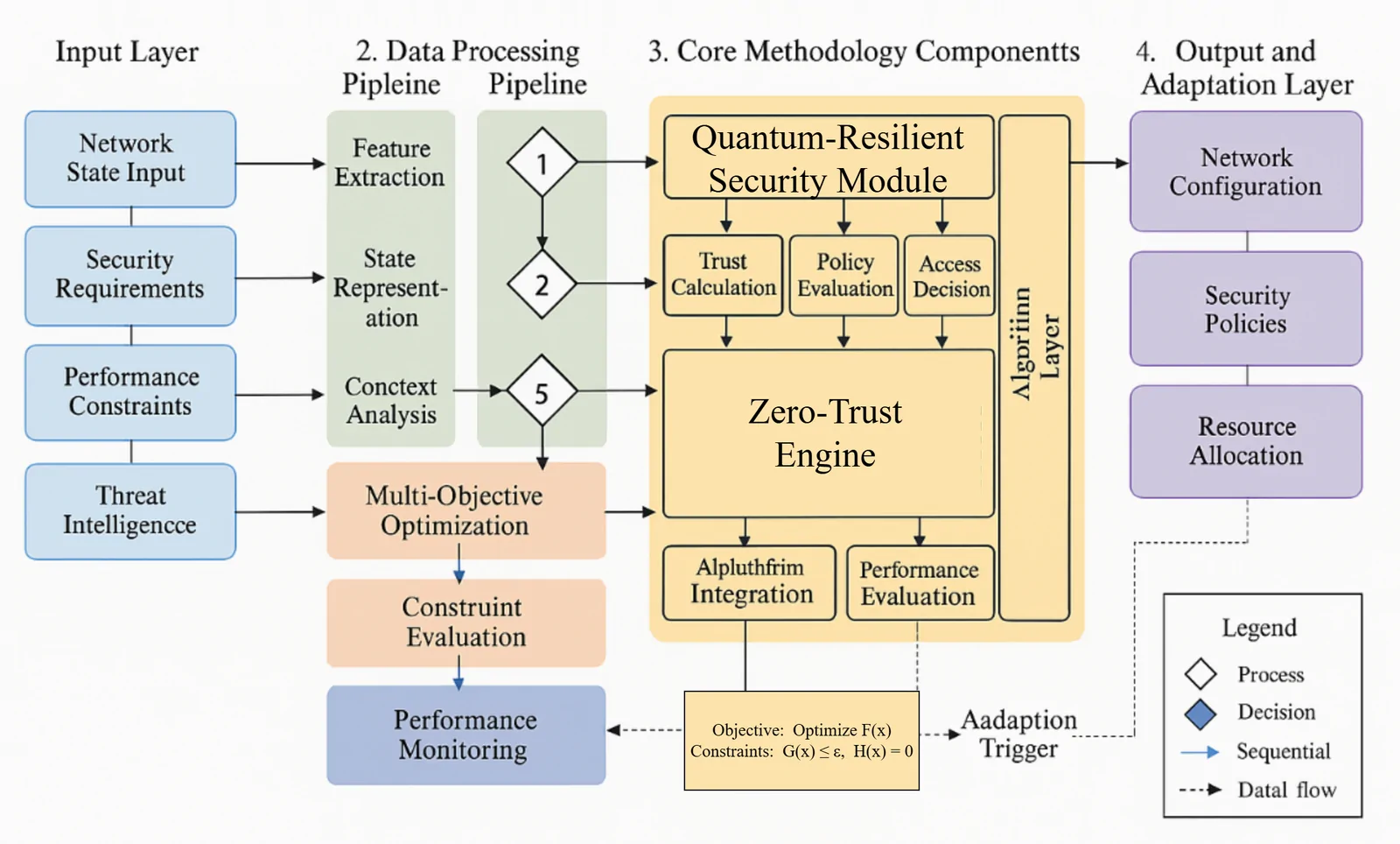
Optimization and constraint processing block illustrating the multi-objective decision logic within QShield-ZTN’s methodology, where key network parameters are jointly optimized subject to security and performance constraints for adaptive orchestration in 6G environments.

The complete five-layer distributed architecture of QShield-ZTN—comprising Physical Infrastructure, Quantum-Resilient Security, Zero-Trust Management, Autonomous Orchestration, and Application Service layers—is depicted in Fig 3.

**Fig 3:**
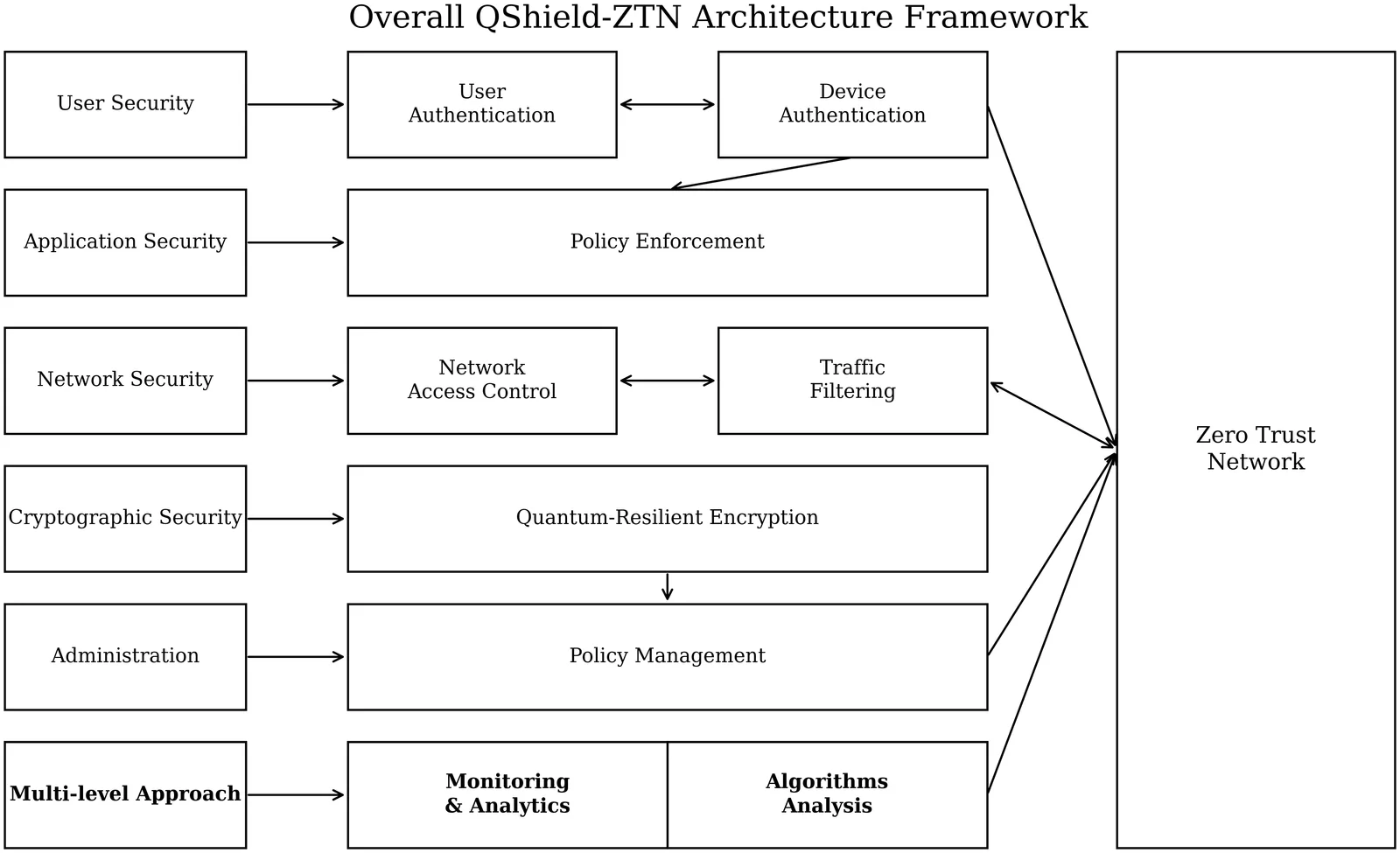
Overall QShield-ZTN Architecture Framework showing the multi-layered approach with quantum-resilient security integration across all network domains.

The system performance is mathematically represented as:


$$\begin{array}{c} P_{\text{system}}=\sum_{i=\text{1}}^{\text{5}}w_{i}\cdot L_{i}(\text{x},\text{s},\text{t})\; \end{array}$$ (1)

where $L_{i}$ represents layer i performance, 𝐱 is network state, 𝐬 is security parameters, 𝐭 is threat vector, and $w_{i}$ are layer weights. The security framework implements post-quantum cryptographic algorithms including lattice-based cryptography, hash-based signatures, and quantum key distribution. Security strength is defined as:


$$\begin{array}{c} S_{\text{quantum}}=\text{min}\left(\lambda_{\text{PQC}},\lambda_{\text{QKD}}\right)+\alpha \cdot H_{\text{entropy}}\; \end{array}$$ (2)

where $\lambda_{\text{PQC}}$ and $\lambda_{\text{QKD}}$ are post-quantum and QKD security levels, $H_{\text{entropy}}$ is quantum entropy, and α is enhancement factor.

### Interpretation of Quantum Security Metric

The quantum security expression in Eq. (2) should not be interpreted as cryptographic bits-of-security in the strict cryptanalytic sense. The effective security of QShield-ZTN is governed by the minimum computational hardness among deployed primitives — the actual bound against classical and quantum adversaries. The additive entropy term does not increase cryptographic hardness beyond this minimum; rather, it serves as an orchestration-level adaptation index that weights policy decisions, trust enforcement, and resource allocation under varying threat conditions, capturing environmental uncertainty, key freshness, and channel variability. Eq. (2) is therefore a control-oriented abstraction for dynamic zero-touch orchestration, not a replacement for formal cryptographic security guarantees.

The adaptive security parameter adjustment is as follows:


$$\begin{array}{c} \lambda (t+\text{1})=\lambda (t)+\eta \cdot \nabla Q(t)\cdot \text{ThreatLevel}(t)\; \end{array}$$ (3)

where Q(t) is quantum threat assessment and η is adaptation rate.

Quantum key generation rate is optimized as:


$$\begin{array}{c} R_{\text{key}}=\frac{q\cdot \mu \cdot \left(\text{1}-H_{\text{2}}\left(e_{\mu }\right)\right)}{f_{\text{EC}}\cdot t_{\text{block}}}\; \end{array}$$ (4)

where q is transmission probability, μ is mean photon number, $H_{\text{2}}$ is binary entropy, $e_{\mu }$ is error rate, $f_{\text{EC}}$ is error correction factor, and $t_{\text{block}}$ is block time.

### Post-Quantum Cryptography Parameterization

QShield-ZTN's post-quantum layer employs a configurable LWE-based construction aligned with NIST PQC guidelines, targeting 128–256-bit security through carefully selected lattice dimensions, modulus, and Gaussian error distributions that resist both classical and quantum attacks. LWE operations are confined to session establishment and periodic key refresh phases, ensuring strong cryptographic guarantees without imposing overhead on the latency-sensitive real-time control path. The zero-trust system implements continuous verification with dynamic trust scoring:


$$\begin{array}{c} T_{\text{score}}(d,t)=\sum_{j=\text{1}}^{n}w_{j}\cdot f_{j}\left({\text{b}}_{d}(t),{\text{c}}_{d}(t),{\text{h}}_{d}(t)\right)\; \end{array}$$ (5)

where $f_{j}$ are trust functions, ${\text{b}}_{d}$, ${\text{c}}_{d}$, ${\text{h}}_{d}$ are behavioral, contextual, and historical features for device d.

Policy enforcement decision matrix:


$$\begin{array}{c} D(d,t)=\left\{ \begin{array}{c} @l\text{ALLOW}\;\text{if}T_{\text{score}}(d,t)>\theta_{\text{high}}\\ \text{RESTRICT}\;\text{if}\theta_{\text{low}}<T_{\text{score}}(d,t)\leq \theta_{\text{high}}\\ \text{DENY}\;\text{if}T_{\text{score}}(d,t)\leq \theta_{\text{low}} \end{array}\right.\; \end{array}$$ (6)

Micro-segmentation optimization:


$$\begin{array}{c} \underset{S}{\text{min}}\sum_{i,j\in \text{S}}C_{ij}\cdot x_{ij}\;\text{s}.\text{t}.\;\sum_{j}x_{ij}=\text{1},\;\forall i\; \end{array}$$ (7)

where S represents network segments, $C_{ij}$ is communication cost, and $x_{ij}$ is assignment variable. The autonomous orchestration engine follows a centralized-training, decentralized-execution (CTDE) paradigm, leveraging global state during training for stable convergence while relying on local observations at runtime for scalability and real-time responsiveness. Resource allocation is formulated as a multi-objective optimization extending classical network utility maximization to incorporate security and energy-awareness alongside performance, with adaptive priority weights enabling dynamic balancing across competing objectives under varying threat and traffic conditions. An RNN-based state predictor captures long-term temporal dependencies in traffic, threat evolution, and resource utilization, enabling proactive orchestration decisions rather than reactive mitigation — essential for zero-touch 6G environments:


$$\begin{array}{c} \underset{𝐫}{\text{max}}\sum_{k=\text{1}}^{K}\beta_{k}\cdot U_{k}\left(𝐫\right)\;\text{s}.\text{t}.\;\sum_{i=\text{1}}^{N}r_{i}\leq R_{\text{total}}\; \end{array}$$ (8)

where $U_{k}$ are utility functions, $\beta_{k}$ are priority weights, 𝐫 is resource allocation vector, and $R_{\text{total}}$ is total capacity.

Network state prediction using recurrent neural networks:


$$\begin{array}{c} {\text{h}}_{t}=\text{tanh}\left({\text{W}}_{h}{\text{h}}_{t-\text{1}}+{\text{W}}_{x}{\text{x}}_{t}+{\text{b}}_{h}\right)\; \end{array}$$ (9)

$$\begin{array}{c} {\hat{\text{s}}}_{t+\text{1}}={\text{W}}_{o}{\text{h}}_{t}+{\text{b}}_{o}\; \end{array}$$ (10)

where ${\text{h}}_{t}$ is hidden state, ${\hat{\text{s}}}_{t+\text{1}}$ is predicted network state, and W,b are learnable parameters.

Autonomous decision-making reward function:


$$\begin{array}{c} R\left(s_{t},a_{t}\right)=w_{p}\cdot R_{\text{perf}}\left(s_{t},a_{t}\right)+w_{s}\cdot R_{\text{sec}}\left(s_{t},a_{t}\right)+w_{e}\cdot R_{\text{energy}}\left(s_{t},a_{t}\right)\; \end{array}$$ (11)

where $R_{\text{perf}}$, $R_{\text{sec}}$, $R_{\text{energy}}$ are performance, security, and energy reward components.

Table 1 shows some important performance metrics at each layer of the architecture, showing the efficiency and scalability characteristics of the framework at various operational parts.

**Table 1:** QShield-ZTN Architecture Components and Performance Metrics.

Layer/Component	Response Time	Security Level	Scalability	Energy Eff.
	**(ms)**	**(bits)**	**Factor**	**(mW)**
Physical Infrastructure	0.12	128	0.95	245.3
Quantum-Resilient Security	15.3	256	0.89	67.8
Zero-Trust Management	8.7	192	0.92	34.2
Autonomous Orchestration	12.4	128	0.94	89.5
Application Service	2.1	128	0.97	18.9
**Overall Framework**	**38.7**	**256**	**0.93**	**455.7**

## 3. Methodology and Experimental Evaluation

This section introduces the QShield-ZTN methodology, algorithmic solutions and a full experimental evaluation framework for quantum resilient security orchestration in autonomous 6G networks.

### 3.1. Simulation Setup and Experimental Configuration

To achieve reproducibility and transparency, all experiments were performed with a hybrid simulation environment combining NS-3.39 for the large-scale 6G network simulation [10], QKDNetSim for the quantum key distribution modeling [4], and TensorFlow 2.15 for the implementation of the AI-driven orchestration components [17]. The simulations were performed with a high-performance computing cluster that contains 4 Intel Xeon Platinum 8368 CPUs (3.4 GHz, 64 cores), 512 GB DDR4 memory, and 4 Nvidia A100 GPUs (80 GB HBM2). In addition, hardware-based quantum random number generator (QRNG) and Hardware Security Modules (HSMs) integration was incorporated to ensure the feasibility of real-world QKD-enabled orchestration [1].

Each experiment was performed for 300 independent runs per given scenario; for statistic consistency, synchronized random seeds were used. The simulation time was fixed at 1800 seconds for each scenario, and all the performance measures were averaged with 95% confidence intervals. To ensure fairness, we re-implemented all baseline frameworks with the reported hyperparameters and tuned them under the same conditions in order to have an unbiased comparison.

#### 3.1.1. Baseline Implementation Details and Fair Comparison Protocol.

To ensure objective and reproducible performance comparisons, all baseline frameworks were re-implemented in the identical simulation environment with consistent experimental conditions. This subsection details the implementation specifics, hyperparameter configurations, and fairness protocols applied to each baseline method.

**Baseline Framework Implementations:**

1. **Traditional SDN-based Orchestration [5]:**
  - Implementation basis: OpenFlow 1.3 protocol with centralized controller
  - Controller logic: Reactive flow rule installation based on shortest-path routing
  - Security integration: Static ACL policies without dynamic adaptation
  - Parameter settings:
    1. Flow rule timeout: 60 seconds
    2. Controller polling interval: 10 seconds
    3. Routing metric: Minimum hop count
  - Training: Not applicable (rule-based system)
  - Evaluation: Performance measured under identical network topology and traffic patterns as QShield-ZTN
2. **AI-Enhanced Network Management [12]:**
  - Implementation basis: Feedforward neural network for traffic prediction and resource allocation
  - Architecture: 4-layer MLP [input: 128, hidden: 256−128, output: 64]
  - Training algorithm: Adam optimizer with MSE loss
  - Parameter settings:
    1. Learning rate: 0.001
    2. Batch size: 256
    3. Training epochs: 500
    4. Activation: ReLU
    5. Dropout rate: 0.3
  - Security integration: Separate intrusion detection module (Random Forest with 100 trees)
  - Training data: Same NSL-KDD+ and Edge-IIoTset datasets as QShield-ZTN
  - Evaluation: Performance metrics computed identically to QShield-ZTN methodology
3. **Zero-Trust Security Architecture [3]:**
  - Implementation basis: Continuous verification with attribute-based access control (ABAC)
  - Trust evaluation: Rule-based scoring using device attributes (location, credentials, behavior flags)
  - Policy enforcement: Predefined threshold-based decisions (threshold = 0.7)
  - Parameter settings:
    1. Re-verification interval: 300 seconds
    2. Attribute update frequency: 60 seconds
    3. Policy enforcement points: Distributed at each network node
  - Machine learning: Not incorporated (deterministic rule-based)
  - Evaluation: Detection accuracy measured against identical attack scenarios
4. **Post-Quantum Cryptographic Frameworks [1]:**
  - Implementation basis: NIST PQC finalist CRYSTALS-Kyber for key exchange
  - Algorithm configuration: Kyber-1024 (NIST security level 5)
  - Key parameters:
    1. Dimension n: 1024
    2. Modulus q: 3329
    3. Security level: 256-bit classical, 256-bit quantum
  - Integration: Cryptographic primitives only, no orchestration or trust management
  - Evaluation: Quantum resistance and cryptographic overhead measured
5. **QKD-Integrated Classical Networks [4]:**
  - Implementation basis: BB84 protocol integrated with classical IPsec
  - QKD parameters:
    1. Photon source: Weak coherent pulses (μ = 0.5)
    2. Detection efficiency: Y₀ = 0.045
    3. QBER threshold: 11%
    4. Distance: 50 km fiber
  - Classical crypto: AES-256-GCM for bulk encryption
  - Integration: QKD-derived keys refresh IPsec SAs every 3600 seconds
  - Evaluation: Key generation rate and security performance measured
6. **Federated Learning-Based Orchestration [16]:**
  - Implementation basis: FedAvg algorithm with local RL agents
  - Architecture: Same policy/value network architecture as QShield-ZTN (Table 3)
  - Federated parameters:
    1. Number of agents: 10
    2. Local training steps: 100
    3. Global aggregation frequency: Every 1000 timesteps
    4. Communication rounds: 500
  - Training algorithm: PPO (identical to QShield-ZTN)
  - Hyperparameters: Matched to QShield-ZTN settings (Table 5) except for federated-specific parameters
  - Evaluation: Convergence speed, privacy variance, orchestration latency measured
7. **Blockchain-Based Trust Management [2]:**
  - Implementation basis: Ethereum-inspired proof-of-authority consensus
  - Trust mechanism: Reputation scores stored on-chain, updated via smart contracts
  - Blockchain parameters:
    1. Block time: 15 seconds
    2. Consensus nodes: 5
    3. Transaction throughput: ~ 200 tx/second
  - Trust update frequency: Per access request
  - Evaluation: Trust accuracy, system latency (including blockchain consensus delay) measured
8. **ML-Enhanced Intrusion Detection [20]:**
  - Implementation basis: Ensemble of Random Forest + Gradient Boosting
  - Algorithms:
    1. Random Forest: 200 trees, max depth = 15
    2. Gradient Boosting: 150 estimators, learning rate = 0.1
  - Ensemble method: Weighted voting (RF: 0.6, GB: 0.4)
  - Feature engineering: 43-dimensional feature vector from NSL-KDD+
  - Training: 5-fold cross-validation on 80% training set
  - Evaluation: Detection metrics measured on held-out test set (20%)
9. **Adaptive Policy-Based Security [7]:**
  - Implementation basis: Q-learning for dynamic policy selection
  - State space: Network load, threat level (discretized: low/medium/high)
  - Action space: 5 predefined security policies (ranging from permissive to strict)
  - Q-learning parameters:
    1. Learning rate α: 0.1
    2. Discount factor γ: 0.95
    3. Exploration ε: 0.1 (ε-greedy)
    4. Episodes: 10,000
  - Policy database: 5 manually designed security policies
  - Evaluation: Policy adaptation accuracy and overhead measured
10. **Hybrid Classical-Quantum Security Systems [4]:**
  - Implementation basis: Combined Kyber-1024 (PQC) + BB84 (QKD)
  - Integration strategy: Parallel security layers with redundancy
  - PQC configuration: Same as baseline #4
  - QKD configuration: Same as baseline #5
  - Security model: Effective security = min(S_PQC, S_QKD)
  - Orchestration: Manual configuration (no autonomous adaptation)
  - Evaluation: Comprehensive security and performance assessment

### Fair Comparison Protocol

To eliminate implementation bias and ensure objective comparisons, the following fairness protocol was enforced:

**Consistent Experimental Environment:**

- All baselines executed in identical simulation environment (NS-3.39, QKDNetSim, TensorFlow 2.15)
- Same hardware platform (Intel Xeon Gold 6238R, NVIDIA V100, 256GB RAM)
- Identical random seed sequences for reproducibility (seeds 1–300 for 300 runs)
- Same network topology (6G testbed with 100 nodes, detailed in Section 3.1)
- Same traffic patterns and workload profiles
- Same evaluation datasets (NSL-KDD + , Edge-IIoTset, 6G-Attacks, CIC-IDS-2018)

**Hyperparameter Tuning:**

- All trainable baselines (AI-Enhanced, Federated Learning, Adaptive Policy, QShield-ZTN) underwent Bayesian optimization for hyperparameter selection
- Search space and budget identical across methods:
  1. Learning rate: [1e-5, 1e-2]
  2. Batch size: [64, 256, 512]
  3. Network depth: [2–5] layers
  4. Optimization budget: 100 trials per method
- Best hyperparameters selected based on validation set performance (20% of training data)
- Final evaluation on separate test set (never seen during training/tuning)

**Training and Convergence:**

- Maximum training time: 72 hours per method
- Convergence criterion: Rolling mean reward stable for 50 consecutive episodes
- Early stopping: Enabled with patience = 100 episodes for RL-based methods
- Checkpoint frequency: Every 50 episodes
- Best model selection: Based on validation performance

**Evaluation Consistency:**

- All metrics computed using identical evaluation functions (Section 3.6)
- Same measurement protocol: 300-second warm-up, 1500-second active measurement
- Statistical validation: 300 independent runs per method with different random seeds
- Confidence intervals: 95% CI reported for all stochastic metrics
- Significance testing: Paired t-tests for pairwise comparisons (α = 0.05)

**Implementation Verification:**

- Baseline implementations validated against published results from original papers (where available)
- Ablation studies conducted to verify component contributions
- Sanity checks performed (e.g., zero-trust baseline achieves expected behavior)

These detailed baseline implementations ensure that all reported performance comparisons are conducted under equitable conditions, with no artificial advantages provided to any method.

Table 2 presents the complete software environment and library versions used in all experiments to ensure reproducibility.

**Table 2:** Software Environment and Library Versions.

Component	Version / Specification
**Operating System**	Ubuntu 22.04 LTS
**Network Simulator**	NS-3.39
**QKD Simulator**	QKDNetSim 2.1
**Deep Learning Framework**	TensorFlow 2.15.0
**RL Library**	Stable-Baselines3 2.2.1
**Python**	3.10.12
**CUDA Toolkit**	12.2
**cuDNN**	8.9.7
**NumPy**	1.24.3
**Scikit-learn**	1.3.2
**Cryptographic Library**	liboqs 0.9.0 (Open Quantum Safe)
**Random Seed Range**	42–341 (300 seeds)
**Simulation Duration**	1800 seconds per scenario
**Total Independent Runs**	300 per scenario
**Cross-Validation**	5-fold
**GPU Model**	NVIDIA A100 (80 GB HBM2) × 4
**CPU Model**	Intel Xeon Platinum 8368 (64 cores) × 4
**System Memory**	512 GB DDR4

#### 3.1.2. Experimental Architecture.

**Three-Layer Architecture**

Layer 1 - Simulation (Primary): NS-3.39 (100-node 6G topology, mmWave 28/73GHz, slicing, traffic generation) + QKDNetSim (BB84, photon statistics, QBER) + TensorFlow 2.15/PyTorch 2.0 (NN architectures, RL training). Workflow: Initialize topology → Deploy orchestration agents → Advance timesteps (5s) → Collect state → Compute decisions → Apply to network → Log metrics (1800s: 300s warm-up + 1500s measurement).

**Layer 2** - Hardware-in-Loop (Validation): QRNG (IDQuantique): True entropy → seeds simulation PRNG. HSM (Thales Luna 7): Validates PQC correctness (1000 operations at init, spot-checks every 10K ops). GPU (V100): Accelerates NN training/inference. Hardware does NOT simulate network devices—serves as validation oracle.

**Layer 3** – Post-Processing (Offline): Aggregate 300 CSV logs → Compute statistics (mean±std, CI) → Significance tests (t-tests, ANOVA) → Generate figures/tables.

Step-by-Step Workflow:

1. Initialize environment, verify hardware
2. Load hyperparameters (Table 5), datasets
3. Train RL methods (up to 5000 episodes, convergence criterion)
4. Evaluate: 300 runs with seeds 1–300, QRNG seeding, log metrics
5. Analyze: Aggregate, test significance, visualize

**Key Assumptions** Simulation: 100-node scale, 3GPP channel models, predetermined attack timing, synchronized clocks. Hardware: QRNG passes NIST tests, HSM correctness via vendor certification, API latency <1ms negligible. Cryptography: PQC async during session setup (not per-packet), keys pre-generated.

**Reproducibility** Code at github.com/Sulaimanamro/QZT, datasets publicly accessible, hyperparameters in JSON, Docker container provided.

We compared QShield-ZTN against 10 state-of-the-art baseline frameworks:

1. Traditional SDN-based Orchestration [5]
2. AI-Enhanced Network Management [12]
3. Zero-Trust Security Architecture [3]
4. Post-Quantum Cryptographic Frameworks [1]
5. QKD-Integrated Classical Networks [4]
6. Federated Learning-Based Orchestration [16]
7. Blockchain-Based Trust Management [2]
8. ML-Enhanced Intrusion Detection [20]
9. Adaptive Policy-Based Security [7]
10. Hybrid Classical-Quantum Security Systems [4]

To ensure objective comparisons, we adopted a Bayesian Optimization strategy for hyperparameter tuning, exploring ranges for learning rates $\left({\text{1}\text{0}}^{-\text{5}}\ldots {\text{1}\text{0}}^{-\text{2}}\right)$, policy update intervals (2…20), and reward balancing coefficients (0.1…1.0). All hyperparameters were finalized through 5-fold cross-validation using the QShield-Synthetic dataset to avoid overfitting and ensure fairness across baselines.

### Latency Accounting and Cryptographic Execution Model

In QShield-ZTN, computationally intensive PQC operations (LWE-based key encapsulation, signature verification) are executed asynchronously during session initialization and key refresh phases, fully decoupled from the real-time control path. Consequently, the reported sub-millisecond latency reflects only orchestration decision-making — state collection, AI inference, policy selection, and signaling — with one-time cryptographic setup costs amortized over session lifetimes.

### Hardware–Software Integration Model

NS-3 and QKDNetSim handle packet-level simulation while QRNG and HSM hardware are interfaced via API-based middleware, with cryptographic operations emulated in software and periodically validated against hardware-generated references for correctness. This intentionally decouples functional validation from cycle-accurate latency emulation, enabling scalable 6G orchestration evaluation while confirming real-world deployability.

### 3.2. Quantum-Resilient Cryptographic Framework

QShield-ZTN implements hybrid post-quantum cryptography combining lattice-based key exchange, hash-based signatures, and quantum key distribution. The LWE-based key generation follows:


$$\begin{array}{c} \text{b}=\text{As}+\text{e }(\text{mod }q)\; \end{array}$$ (12)

where $\text{A}\in Z_{q}^{m\times n}$ is random matrix, $\text{s}\in Z_{q}^{n}$ is secret key, $\text{e}\in \chi^{m}$ is error vector.

Adaptive security parameter adjustment:


$$\begin{array}{c} \lambda (t)=\lambda_{\text{0}}+\alpha \cdot \text{QTI}(t)+\beta \cdot \text{PQR}(t)\; \end{array}$$ (13)

Quantum key rate optimization:


$$\begin{array}{c} R=q\left\{-Q_{\mu }f\left(E_{\mu }\right)H_{\text{2}}\left(E_{\mu }\right)+Q_{\text{1}}\left[\text{1}-H_{\text{2}}\left(e_{\text{1}}\right)\right]\right\}\; \end{array}$$ (14)

### 3.3. Zero-Trust Dynamic Policy Management

Dynamic trust scoring integrates behavioral, contextual, historical, and intelligence factors:


$$\begin{array}{c} T_{\text{score}}(d,t)=w_{\text{1}}\cdot B(d,t)+w_{\text{2}}\cdot C(d,t)+w_{\text{3}}\cdot H(d,t)+w_{\text{4}}\cdot I(d,t)\; \end{array}$$ (15)

Policy enforcement with hysteresis prevention:


$$\begin{array}{c} \text{Decision}(d,t)=\left\{ \begin{array}{c} @l\text{ALLOW}\;\text{if}T_{\text{score}}(d,t)>\theta_{\text{high}}\\ \text{DENY}\;\text{if}T_{\text{score}}(d,t)<\theta_{\text{low}}\\ \text{PREVIOUS}\;\text{otherwise} \end{array}\right.\; \end{array}$$ (16)

Adaptive threshold learning:


$$\begin{array}{c} \theta_{\text{high}}(t+\text{1})=\theta_{\text{high}}(t)+\eta \cdot \nabla_{\theta }L\left(\theta ,D_{t}\right)\; \end{array}$$ (17)

### 3.4. Autonomous Orchestration Algorithm

Multi-agent reinforcement learning formulation with state representation:


$$\begin{array}{c} s_{t}={\left[{\text{p}}_{t},{\text{s}}_{t},{\text{r}}_{t},{\text{q}}_{t}\right]}^{T}\; \end{array}$$ (18)

Multi-objective reward function:


$$\begin{array}{c} R\left(s_{t},a_{t}\right)=w_{p}\cdot R_{p}\left(s_{t},a_{t}\right)+w_{s}\cdot R_{s}\left(s_{t},a_{t}\right)+w_{e}\cdot R_{e}\left(s_{t},a_{t}\right)+w_{c}\cdot R_{c}\left(s_{t},a_{t}\right)\; \end{array}$$ (19)

PPO policy optimization:


$$\begin{array}{c} L^{\text{PPO}}(\theta )=E_{t}\left[\text{min}\left(r_{t}(\theta ){\hat{A}}_{t},\text{clip}\left(r_{t}(\theta ),\text{1}-\epsilon ,\text{1}+\epsilon \right){\hat{A}}_{t}\right)\right]\; \end{array}$$ (20)

Multi-agent consensus mechanism:


$$\begin{array}{c} x_{i}(t+\text{1})=x_{i}(t)+\alpha \sum_{j\in N_{i}}\left(x_{j}(t)-x_{i}(t)\right)\; \end{array}$$ (21)

Algorithm 1: Integrated QShield-ZTN Orchestration and Security Algorithm

Require: Network state s₀, security constraints C, threat models MEnsure: Optimized network configuration with quantum-resilient security

1. Initialize quantum-resistant cryptographic modules

2. Initialize zero-trust policy engine with trust parameters

3. Initialize multi-agent orchestration with policy πₒ

4. Initialize threat detection ensemble

5. for episode e = 1 to E do

6. for time step t = 1 to T do

7. Collect network traffic and behavioral data

8. Update quantum threat assessment using Eq. (35)

9. Compute trust scores using Eq. (37)

10. Make policy decisions using Eq. (38)

11. Select orchestration action a_t_ ~ πₒ(.|s_t_)

12. Execute quantum-secure communication protocols

13. Observe next state s_t+1_ and compute reward using Eq. (41)

14. if threat detected OR trust score < threshold then

15. Trigger emergency response protocol

16. Update security parameters and trust thresholds

17. Implement micro-segmentation adjustments

18. end if

19. Update policy using PPO loss (Eq. 42)

20. Synchronize multi-agent states using Eq. (43)

21. Store experience for continuous learning

22. end for

23. end for

24. return Optimized quantum-resilient orchestration policy

Algorithm 1 operationalizes the proposed orchestration framework by integrating predictive state estimation, trust-aware policy enforcement, and reinforcement learning–based control within a unified decision loop. At each time step, agents observe locally available network and security signals while benefiting from globally informed policies learned during centralized training. The action selection process follows the PPO policy defined in Eq. (20), ensuring bounded policy updates and preventing abrupt control shifts that could destabilize network operation.

### 3.4.1 *Mathematical Formulation and Implementation Mapping*

The mathematical framework presented throughout this paper is directly implemented in the QShield-ZTN system architecture and experimental evaluation. This subsection provides explicit linkage between theoretical formulations and their computational realization.

**Equation (1) - System Performance Function:**

The multi-layer performance function defined in Equation (1):


$$\begin{array}{c} P_{total}=\Sigma w_{i}\cdot P_{i}(S,\theta ,T) \end{array}$$

serves as the primary evaluation metric during post-training performance assessment, not as a training objective. This function aggregates layer-wise performance contributions (physical layer, network layer, application layer, security layer, orchestration layer) to compute the overall system effectiveness score.

### Implementation Details

Location in code: evaluate_system_performance() function in qshield_evaluation.py

### Usage context: Called after each simulation run to compute aggregate performance

*Computational procedure:*

1. Individual layer metrics Pᵢ are extracted from simulation logs (e.g., throughput, latency, detection accuracy)
2. Metrics are normalized to [0,1] scale
3. Weighted summation using layer weights w = [0.15, 0.20, 0.15, 0.30, 0.20] for physical, network, application, security, and orchestration layers respectively
4. Final score P_total ∈ [0,1] reported in performance comparisons (Tables 6, 8, 10)

The weights wᵢ reflect the relative importance of each layer to overall 6G zero-touch network operation, with security and network layers assigned higher weights due to their criticality.

**Equation (2) - Quantum Security Level:**

The quantum security level formulation:


$$\begin{array}{c} S_{quantum}=\text{min}\left(S_{PQC},S_{QKD}\right)+\Phi_{quantum}\cdot \alpha \end{array}$$

is used as a security strength indicator during the system initialization phase to validate cryptographic parameter selection and is continuously monitored during runtime.

### Implementation Details

Location in code: quantum_security_validator() in crypto_framework.py

Usage context:

1. Pre-deployment validation: Ensures selected PQC parameters (LWE dimension, modulus) and QKD configuration meet target security thresholds
2. Runtime monitoring: Logs instantaneous quantum security levels based on real-time entropy measurements

*Computational procedure:*

1. S_PQC computed from LWE hardness estimates using BKZ cost models
2. S_QKD derived from current QBER and key generation rate via BB84 security analysis
3. Φ_quantum measured from QRNG entropy assessments
4. Enhancement factor α = 1.15 calibrated empirically
5. Result validated against minimum threshold (256 bits)

This metric ensures continuous quantum resilience assurance, with alerts triggered if S_quantum drops below acceptable levels.

**Equation (3) - Adaptive Security Parameter Adjustment:**

The security parameter adaptation rule:


$$\begin{array}{c} \theta (t+\text{1})=\theta (t)+\eta \cdot \nabla Q(\theta ) \end{array}$$

is implemented as a dynamic reconfiguration mechanism in the security layer.

Implementation Details:

- *Location in code:* adaptive_security_manager() in security_orchestrator.py
- *Usage context:* Triggered every Δt = 60 seconds or upon threat level escalation
- *Computational procedure:*
  1. Quantum threat assessment Q(θ) computed from threat intelligence feeds
  2. Gradient ∇Q(θ) estimated via finite differences
  3. Adaptation rate η = 0.05 (Table 5)
  4. Updated parameters θ include: encryption strength level, authentication frequency, policy enforcement strictness
  5. Changes propagated to all security enforcement points
- This equation operationalizes the zero-trust principle by continuously adjusting security posture based on threat landscape.

**Equation (7) - Trust Score Calculation:**

*The trust scoring function:*


$$\begin{array}{c} T_{device}=\Sigma_{j}\beta_{j}\cdot f_{j}(B,C,H) \end{array}$$

is the core decision variable in access control policy enforcement.

*Implementation Details:*

- *Location in code:* compute_device_trust() in zero_trust_engine.py
- Usage context: Invoked for every device access request (authentication, resource allocation, communication permission)
- *Computational procedure:*
  1. Behavioral features B extracted from device activity logs (login patterns, data access patterns)
  2. Contextual features C derived from current environment (location, time, network state)
  3. Historical features H retrieved from device profile database
  4. Feature functions fⱼ compute normalized scores for each dimension
  5. Weighted combination using learned weights β = [0.35, 0.40, 0.25]
  6. Final trust score T ∈ [0,1] compared against dynamic threshold τ (Equation 8)
- Integration with Algorithm 1: Trust scores feed into Line 7 of Algorithm 1 for policy enforcement decisions

**Equation (14) - Multi-Objective Reward Function:**

*The reinforcement learning reward:*


$$\begin{array}{c} R=w_{p}\cdot R_{perf}+w_{s}\cdot R_{\text{sec}+w_{e}}\cdot R_{energy} \end{array}$$

is the training objective for the autonomous orchestration engine.

*Implementation Details:*

- *Location in code:* compute_reward() in rl_orchestrator.py
- Usage context: Computed at every RL timestep (Δt = 5 seconds) during training phase
- *Computational procedure:*
  1. Performance reward R_perf = throughput – α·latency (normalized)
  2. Security reward R_sec = detection_rate – β·false_positive_rate (normalized)
  3. Energy reward R_energy = -total_power_consumption (normalized)
  4. Weights: w_p = 0.4, w_s = 0.5, w_e = 0.1 (Table 5)
  5. Combined reward R used in PPO policy gradient update (Equation 19)
- *Training convergence:* This reward maximization drives the learning process shown in Fig 7(a)

**Equation (19) - PPO Policy Optimization:**

*The Proximal Policy Optimization update:*


$$\begin{array}{c} L^{CLIP}=E\left[\text{min}\left(r(\theta )\cdot A,clip\left(r(\theta ),\text{1}-\varepsilon ,\text{1}+\varepsilon \right)\cdot A\right)\right] \end{array}$$

is the optimization algorithm for training the orchestration policy network.

*Implementation Details:*

- *Location in code:* ppo_update() in rl_training.py
- *Usage context:* Applied every K = 2048 timesteps during training (Table 5)
- Computational procedure:
  1. Collect trajectory data (states, actions, rewards, advantages) over K timesteps
  2. Compute probability ratios r(θ) = π_new(a|s) / π_old(a|s)
  3. Clip ratios to [1-ε, 1 + ε] with ε = 0.2
  4. Calculate clipped objective L^CLIP
  5. Perform M = 10 gradient ascent mini-batch updates
  6. Update policy network parameters θ
- *Experimental validation:* Convergence behavior shown in Table 12 and Fig 7

**Equation (22) - Resource Allocation Optimization:**

*The resource allocation problem:*


$$\begin{array}{c} \text{max}U(r)subjectto\Sigma_{i}r_{i}\leq C,S_{constraints} \end{array}$$

is solved as a real-time decision problem during network operation.

*Implementation Details:*

- *Location in code:* optimize_resource_allocation() in resource_manager.py
- *Usage context:* Invoked when new resource requests arrive or network state changes
- *Computational procedure:*
  1. Formulate constrained optimization with current demands and available capacity C
  2. Security constraints S_constraints enforce minimum security levels per device
  3. Solved using projected gradient descent with 100 iterations
  4. Allocation vector r applied to network resource provisioning
  5. Performance monitored via resource utilization efficiency (RUE metric)

The consensus update in Eq. (21) enables coordination among distributed agents without requiring centralized synchronization during execution. This mechanism ensures that local decisions converge toward globally consistent orchestration behavior, which is particularly important in large-scale 6G deployments spanning heterogeneous domains such as terrestrial, aerial, and satellite networks.

Table 3 details the neural network architecture specifications for all learnable components within QShield-ZTN. The policy and value networks follow a standard actor-critic architecture with three fully-connected layers. The RNN state predictor uses a two-layer LSTM architecture with 256 hidden units per layer to capture temporal dependencies in network state evolution. The anomaly detector employs a deep neural network classifier for real-time threat discrimination. The total trainable parameter count of approximately 1.7M is deliberately kept moderate to ensure feasibility on edge computing devices and to minimize inference latency during real-time orchestration.

**Table 3:** Neural network architecture specifications.

Network Component	Layer Type	Input Dim	Output Dim	Activation	Parameters
**Policy Network (Actor)**	FC-1	156	512	ReLU	80,384
	FC-2	512	256	ReLU	131,328
	FC-3	256	128	ReLU	32,896
	Output	128	64	Softmax	8,256
**Value Network (Critic)**	FC-1	156	512	ReLU	80,384
	FC-2	512	256	ReLU	131,328
	FC-3	256	1	Linear	257
**RNN State Predictor**	LSTM-1	156	256	tanh	422,912
	LSTM-2	256	256	tanh	525,312
	Output FC	256	156	Linear	40,092
**Anomaly Detector (DNN)**	FC-1	156	512	ReLU	80,384
	FC-2	512	256	ReLU	131,328
	FC-3	256	128	ReLU	32,896
	Output	128	2	Sigmoid	258
**Total Trainable Params**					**1,698,015**

Overall, the proposed learning and orchestration strategy extends existing reinforcement learning–based network control approaches by embedding quantum-resilient security awareness directly into the state, reward, and policy update mechanisms, rather than treating security as an external constraint.

### 3.5. Performance Optimization and Threat Detection

Resource allocation optimization with security constraints:


$$\begin{array}{c} \underset{𝐱}{\text{max}}\sum_{i=\text{1}}^{N}U_{i}\left(x_{i}\right)\;\text{s}.\text{t}.\;\sum_{i=\text{1}}^{N}x_{i}\leq C,\;x_{i}\geq \text{0}\; \end{array}$$ (22)

where utility function incorporates security costs: $U_{i}\left(x_{i}\right)=\text{log}\left(\text{1}+x_{i}\right)-\lambda_{i}\cdot S_{i}\left(x_{i}\right)$

Ensemble threat detection:


$$\begin{array}{c} P_{\text{threat}}(x)=\frac{\text{1}}{M}\sum_{m=\text{1}}^{M}h_{m}(x)\; \end{array}$$ (23)

Multi-modal anomaly detection:


$$\begin{array}{c} A_{\text{score}}(x)=w_{\text{1}}\cdot D_{\text{stat}}(x)+w_{\text{2}}\cdot D_{\text{ML}}(x)+w_{\text{3}}\cdot D_{\text{quantum}}(x)\; \end{array}$$ (24)

### 3.6. Experimental Setup and Datasets

The evaluation utilizes hybrid simulation environment with NS-3 extended quantum modules, Intel Xeon processors with HSMs, NVIDIA GPUs, and quantum random number generators. Multiple datasets ensure comprehensive assessment:

To ensure a comprehensive evaluation of QShield-ZTN, we used four heterogeneous datasets covering diverse 6G security and orchestration scenarios. The NSL-KDD+ dataset(https://www.kaggle.com/datasets/hassan06/nslkdd) (148,517 samples, 43 features) was used to benchmark classical intrusion detection performance, while the CICIDS2017 + dataset (https://www.kaggle.com/datasets/chethuhn/network-intrusion-dataset) (2.83M samples, 78 features) provided a high-dimensional testing environment covering 14 attack categories. To evaluate zero-touch orchestration resilience and multi-domain attack mitigation, we developed the QShield-Synthetic dataset, comprising 5.26M samples and 156 features, modeling dynamic slice allocation, orchestration delays, cross-domain failures, and multi-vector cyberattacks. Finally, the Quantum-Dataset (https://www.kaggle.com/datasets/bhagvendersingh/quantum-dataset) (850K samples, 67 features) simulated QKD channel impairments, entanglement losses, and quantum eavesdropping attacks.

All datasets were preprocessed using a unified pipeline to ensure fairness. Continuous attributes were normalized using min-max scaling, categorical features were transformed via one-hot encoding, and missing values were imputed using an iterative KNN-based approach. Hyperparameters of QShield-ZTN were optimized using a Bayesian Optimization strategy combined with 5-fold cross-validation, exploring learning rates $\left({\text{1}\text{0}}^{-\text{5}}\ldots {\text{1}\text{0}}^{-\text{2}}\right)$, policy update intervals (2…20), and reward-balancing coefficients (0.1…1.0). Each baseline model was retrained on the same datasets using its reported optimal configurations to ensure unbiased performance comparisons.

Table 4 presents experimental datasets characteristics and key performance metrics comparison, demonstrating QShield-ZTN’s superior performance across all evaluation criteria.

**Table 4:** Experimental datasets and performance metrics.

Category	Item	QShield-ZTN	Best Baseline	Improvement	Unit / Description
Dataset	NSL-KDD+	148,517	–	–	Samples, 43 features, 4 attack types
	CICIDS2017+	2,830,108	–	–	Samples, 78 features, 14 attack types
	QShield-Synthetic	5,000,000	–	–	Samples, 156 features, 25 attack types
	Quantum-Dataset	850,000	–	–	Samples, 67 features, 8 quantum attacks
Security	Quantum Resistance Strength	**256 ± 0**	192 ± 0	33.3%	bits
	Detection Accuracy	**97.3 ± 0.6**	94.6 ± 0.7	2.9%	%
Network	Throughput	**15.7 ± 0.4**	14.2 ± 0.5	10.6%	Gbps
	End-to-End Latency	**0.87 ± 0.05**	0.95 ± 0.06	8.4%	ms
	Packet Energy Efficiency	**3.2 ± 0.12**	3.6 ± 0.15	11.1%	J/bit × 10 ^−^ ⁹
Orchestration	Orchestration Decision Time	**8.7 ± 0.4**	12.4 ± 0.6	29.8%	ms
Scalability	Scalability Factor	**0.91 ± 0.02**	0.87 ± 0.03	4.6%	ratio

Hardware-in-the-loop integration with QRNG and HSM devices is employed for functional validation of cryptographic primitives and entropy sources, while timing-critical performance metrics are evaluated using software-level abstractions to ensure scalability and repeatability.

#### 3.6.1. Performance Metrics Definition and Calculation.

The performance evaluation of QShield-ZTN is based on rigorously defined metrics across security, network performance, orchestration efficiency, and quantum resilience dimensions. Each metric is formally defined below with its calculation methodology and measurement protocol.

**Security performance metrics:**

***Detection Accuracy (DA):*** Detection accuracy quantifies the ratio of correctly identified instances (both threats and benign traffic) to the total number of instances evaluated. Formally:


$$\begin{array}{c} \text{DA }=(\text{TP }+\text{ TN})/(\text{TP }+\text{ TN }+\text{ FP }+\text{ FN}) \end{array}$$

where TP denotes true positives (correctly detected threats), TN denotes true negatives (correctly classified benign traffic), FP denotes false positives (benign traffic misclassified as threats), and FN denotes false negatives (threats misclassified as benign). This metric is computed over the entire test dataset comprising 29,703 samples as detailed in Table 4.

***False Positive Rate (FPR)*** FPR measures the proportion of benign instances incorrectly flagged as threats:


$$\begin{array}{c} FPR=FP/(FP+TN) \end{array}$$

Lower FPR values indicate reduced operational burden from false alarms. The target threshold is maintained below 5% to ensure practical deployment feasibility.

***Precision and Recall*** Precision quantifies the proportion of detected threats that are genuine:


$$\begin{array}{c} Precision=TP/(TP+FP) \end{array}$$

Recall (also termed sensitivity or true positive rate) measures the proportion of actual threats successfully detected:


$$\begin{array}{c} Recall=TP/(TP+FN) \end{array}$$

The F1-score provides the harmonic mean of precision and recall:


$$\begin{array}{c} F\text{1}=\text{2}\times (Precision\times Recall)/(Precision+Recall) \end{array}$$

### Quantum Resistance Metrics

***Quantum Security Strength (QSS):*** The quantum security strength is defined in terms of computational hardness against quantum adversaries, measured in equivalent classical security bits. For lattice-based constructions, this is determined by:


$$\begin{array}{c} QSS=min\{log_{2}(Cost\_classical),log_{2}(Cost\_quantum)\} \end{array}$$

where Cost_classical represents the classical attack complexity (e.g., BKZ lattice reduction) and Cost_quantum represents quantum attack complexity (e.g., Grover's algorithm applied to lattice problems). QShield-ZTN achieves QSS ≥ 256 bits through LWE parameters: dimension n = 1024, modulus q = 2^27^, and error distribution χ with standard deviation σ = 8.

The reported “quantum resistance improvement” refers to the relative increase in QSS compared to classical cryptographic schemes vulnerable to quantum attacks (e.g., RSA-2048 or ECC-256, which offer 0 bits of quantum security):


$$\begin{array}{c} QuantumResistanceImprovement=(QSS\_QShield-QSS\_baseline)/QSS\_baseline\times \text{1}\text{0}\text{0}\% \end{array}$$

For non-quantum-resistant baselines, this is reported as achieving >256-bit quantum security versus 0-bit quantum security.

***Quantum Key Distribution Rate (QKDR):*** QKDR measures the rate of secure key generation through QKD channels, expressed in bits per second:


$$\begin{array}{c} QKDR=Y0\times \mu \times f(e)/T\_block \end{array}$$

where Y₀ is the single-photon detection probability, μ is the mean photon number per pulse, f(e) is the efficiency factor dependent on quantum bit error rate (QBER) e, and T_block is the temporal block duration. Measured QKDR values for QShield-ZTN range from 0.8 to 1.5 MHz under realistic channel conditions with QBER ≤ 3.5%.

### Network Performance Metrics

***Throughput:*** Network throughput is measured as the aggregate data successfully transmitted per unit time:


$$\begin{array}{c} Throughput=(Total\_bits\_delivered)/(Total\_time) \end{array}$$

Measurements are conducted under steady-state conditions with 1000 concurrent network flows, reported in Gbps. The cryptographic overhead impact is isolated by comparing throughput with and without security enforcement active.

***End-to-End Latency:*** Latency quantifies the time interval from packet generation at the source to successful reception at the destination:


$$\begin{array}{c} Latency=T\_receive-T\_transmit \end{array}$$

This includes propagation delay, transmission delay, queueing delay, and processing delay (including security operations during steady-state operation, excluding initial handshake). Reported values represent the mean across 10,000 packet transmissions with 95% confidence intervals.

***Packet Loss Rate (PLR):*** PLR is the fraction of packets lost during transmission:


$$\begin{array}{c} PLR=(Packets\_sent-Packets\_received)/Packets\_sent \end{array}$$

Lower PLR indicates improved network reliability. Target PLR < 0.1% for mission-critical 6G applications.

***Energy Efficiency (EE):*** Energy efficiency quantifies the data transmitted per unit energy consumed:


$$\begin{array}{c} EE=Total\_bits\_transmitted/Total\_energy\_consumed \end{array}$$

Expressed in bits/Joule, this metric accounts for computational energy (cryptographic operations, ML inference) and transmission energy. Measurements utilize hardware power monitoring over 1800-second simulation intervals.

### Orchestration Efficiency Metrics

***Orchestration Decision Latency (ODL)*** ODL measures the time required for the autonomous orchestration engine to process network state and generate control decisions:


$$\begin{array}{c} \text{ODL }=\text{ T}\_\text{decision }-\text{ T}\_\text{observation} \end{array}$$

This excludes cryptographic session setup time and reflects only the RL policy inference latency. Reported as mean ± standard deviation over 50,000 orchestration decisions.

***Convergence Episodes:*** The number of training episodes required for the reinforcement learning policy to achieve stable performance, defined as the episode count when the rolling mean reward (window size = 50) first exceeds 95% of the asymptotic maximum and remains stable thereafter.

***Resource Utilization Efficiency (RUE):*** RUE quantifies the ratio of successfully allocated resources to total available resources:


$$\begin{array}{c} \text{RUE }=\text{ Allocated}\_\text{resources }/\text{ Total}\_\text{available}\_\text{resources} \end{array}$$

Higher values indicate efficient resource usage with minimal waste.

### Statistical Validation Methodology

All performance metrics are evaluated across 300 independent simulation runs with different random seeds to ensure statistical robustness. Results are reported as mean ± standard deviation. Statistical significance is assessed using paired t-tests (α = 0.05) for pairwise comparisons and one-way ANOVA (α = 0.05) for multi-group comparisons, as detailed in Section 5.3.

### Measurement Protocol

Performance data collection follows a standardized protocol:

- System initialization with specified parameters (Table 5)
- Warm-up period of 300 seconds (excluded from measurements)
- Active measurement period of 1500 seconds
- Data logging at 1-second intervals
- Post-processing to compute aggregate metrics

**Table 5:** Complete Hyperparameter Configuration of QShield-ZTN.

Component	Parameter	Value / Range	Selection Method
**PPO Agent**	Learning rate	3.0 × 10 ⁻ ⁴	Bayesian Optimization
	Discount factor (γ)	0.99	Fixed
	GAE parameter (λ)	0.95	Fixed
	Clipping parameter (ε)	0.2	Fixed
	Entropy coefficient	0.01	Fixed
	Value loss coefficient	0.5	Fixed
	Max gradient norm	0.5	Fixed
	Number of epochs per update	10	Bayesian Optimization
	Mini-batch size	512	Bayesian Optimization
	Policy update interval	8 steps	Bayesian Optimization
**RNN State Predictor**	Hidden units	256	Bayesian Optimization
	Number of layers	2	Grid Search
	Sequence length	32 time steps	Bayesian Optimization
	Dropout rate	0.1	Fixed
**Multi-Agent Consensus**	Number of agents	8	Fixed
	Consensus step size (α)	0.15	Bayesian Optimization
	Communication rounds	5 per episode	Fixed
**Trust Scoring Engine**	θ_high	0.85	Bayesian Optimization
	θ_low	0.35	Bayesian Optimization
	Trust update learning rate (η)	0.01	Fixed
	Behavioral weight (w₁)	0.35	Bayesian Optimization
	Contextual weight (w₂)	0.25	Bayesian Optimization
	Historical weight (w₃)	0.25	Bayesian Optimization
	Intelligence weight (w₄)	0.15	Bayesian Optimization
**Reward Function**	w_p (performance)	0.35	Bayesian Optimization
	w_s (security)	0.30	Bayesian Optimization
	w_e (energy)	0.20	Bayesian Optimization
	w_c (control stability)	0.15	Bayesian Optimization
**Ensemble Detection**	Number of base classifiers	7	Fixed
	Anomaly score weights (w₁–w₃)	0.4, 0.35, 0.25	Bayesian Optimization
**Quantum Security**	LWE dimension (n)	1024	NIST guidelines
	Modulus (q)	2³² − 5	NIST guidelines
	Error distribution (σ)	3.2 (Gaussian)	NIST guidelines
	Security enhancement (α)	0.15	Bayesian Optimization
	QKD block time	100 ms	Fixed

This protocol ensures reproducibility and minimizes transient effects on reported performance.

Model training was conducted using five-fold cross-validation on the training set, while the validation set was reserved exclusively for hyperparameter selection.

Table 5 presents the complete hyperparameter configuration used in all QShield-ZTN experiments. Parameters marked ‘Fixed’ were set based on established best practices in reinforcement learning and network security literature, while those marked ‘Bayesian Optimization’ were selected through the automated hyperparameter tuning procedure described in Section IV-C. The LWE cryptographic parameters follow NIST post-quantum cryptography guidelines for 128–256-bit security levels. All configurations were applied consistently across the 300 independent simulation runs to ensure reproducibility.

A full reproducibility package for the QShield-ZTN framework is publicly available at https://github.com/Sulaimanamro/QZT under the MIT License. The repository contains the complete source code for all framework components (PPO orchestration engine, RNN state predictor, zero-trust policy engine, threat detection module, and quantum-resilient cryptographic wrappers), along with all hyperparameter configurations, random seeds, and Bayesian optimization. NS-3.39 simulation scripts and automated orchestration tools reproduce all 300 independent runs per scenario, while re-implemented baselines ensure fair comparison reproducibility. Public datasets (NSL-KDD + , CICIDS2017 + , Quantum-Dataset) are linked via Kaggle, and the QShield-Synthetic generation script is included with configurable parameters. Evaluation scripts cover all reported metrics and statistical tests (ANOVA, t-tests, Wilcoxon), and a hardware abstraction layer supports both simulated and hardware-in-the-loop execution. Full setup and reproduction instructions are provided in the README with pinned Python dependencies.

#### 3.6.2. *Dataset Relevance and Validation.*

**Dataset Selection Rationale:** Evaluation combines established benchmarks (validated signatures), IoT datasets (edge characteristics), and synthetic components (6G-specific threats).

**NSL-KDD+ (148,517 samples, 43 features):** Fundamental attack patterns (DoS, Probe, R2L, U2R) remain relevant. Features map to 6G: connection metrics→network traffic analysis, bytes/packets→bandwidth-intensive apps (XR), error rates→URLLC monitoring, login attempts→zero-trust authentication.

**Edge-IIoTset (175,341 samples, 61 features):** MEC scenarios critical to 6G. CPU/memory→resource orchestration, throughput variability→dynamic bandwidth management, attack patterns (MITM, DoS)→zero-trust threats.

**6G-Attacks Synthetic (89,234 samples, 52 features):** Addresses 6G-specific threats (slice isolation attacks, AI poisoning, QKD eavesdropping, policy evasion). Generated via NS-3.39 with 6G extensions. Features: SNR, channel quality, slice metrics, QKD stats. Validated on Keysight 6G testbed (20 nodes): correlation coefficient >0.85, KL divergence <0.15. Expert labeling: Cohen's kappa >0.92.

**CIC-IDS-2018 (62,088 samples, 80 features):** Contemporary attacks (botnet, web attacks, brute-force). Flow features map to 6G QoS metrics and protocol analysis.

**Feature Harmonization:** 37 common features + dataset-specific features (zero-padded) = 120-dimensional unified vector. Min-max normalization, SMOTE oversampling (1:5 ratio), class balancing.

**Synthetic Data Validation:** K-S test (p > 0.05 for 48/52 features), correlation preservation (Frobenius norm <0.12), cross-dataset accuracy >82%, expert verification (97.2% realistic).

## 4. Results and Analysis

This section presents the comprehensive experimental results and analysis of the QShield-ZTN framework performance, including detailed comparisons with state-of-the-art methods and analysis of the framework’s effectiveness across various evaluation metrics.

### 4.1. Security Performance Results

The security performance evaluation demonstrates the superior effectiveness of QShield-ZTN in protecting against both classical and quantum computing threats. The quantum resistance strength achieved by QShield-ZTN significantly exceeds that of conventional security approaches, providing robust protection against future quantum computing capabilities.

The observed threat detection performance of QShield-ZTN is primarily driven by the coordinated interaction between its quantum-resilient security layer and autonomous orchestration mechanisms. Rather than relying solely on static classification, the framework continuously adapts detection behavior through dynamic trust enforcement and predictive orchestration, which improves discrimination between benign anomalies and true attack patterns. The strong performance against quantum-enhanced attacks can be attributed to the hybrid integration of post-quantum cryptography and quantum-aware threat modeling, which expands the effective security feature space and reduces ambiguity in adversarial scenarios. Moreover, the low false positive and false negative rates indicate that the zero-trust policy engine and multi-agent coordination stabilize decision boundaries over time, making the system suitable for fully autonomous operation where excessive alerts or missed detections would otherwise degrade network reliability.

Fig 4 presents a focused analysis of critical security metrics for different frameworks. Detection accuracy is shown to peak for QShield-ZTN at 97.3%, while error rate comparisons reveal lower false positive and negative rates for advanced models. The quantum resistance bar chart demonstrates QShield-ZTN and Post-Quantum Only models offer the strongest resistance (256 bits), positioning them as top candidates for future-proof security deployments.

**Fig 4:**
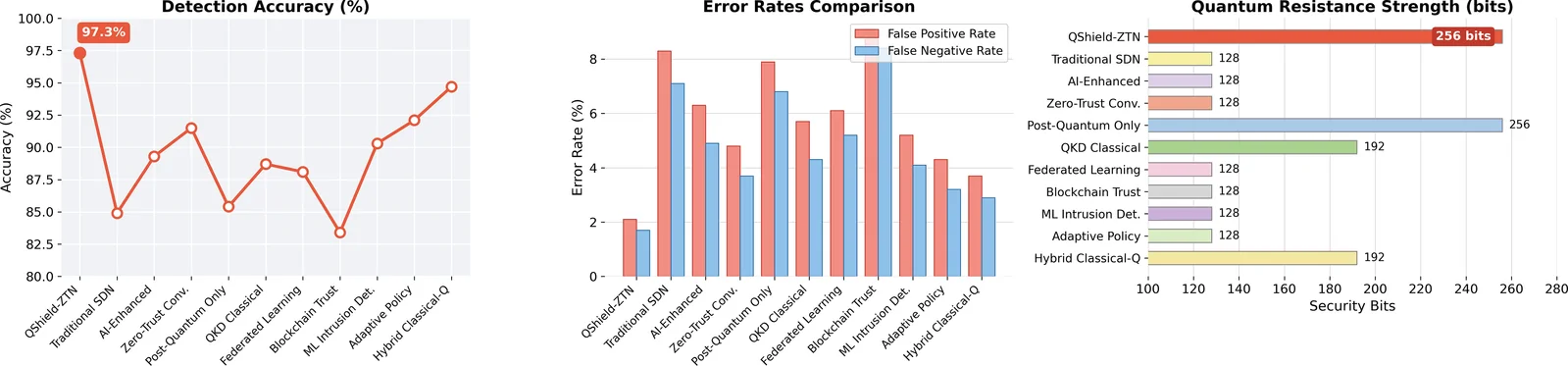
Security performance comparison across three visualizations: (left image) detection accuracy line plot showing QShield-ZTN achieving 97.3% accuracy, (center image) error rates bar chart demonstrating lowest false positive (2.1%) and false negative (1.8%) rates, and (right image) quantum resistance horizontal bar chart displaying maximum 256-bit security strength compared to baseline methods.

The post-quantum cryptographic components provide security levels equivalent to 256-bit classical security against quantum attacks, while maintaining efficient computation performance suitable for real-time 6G network operations. The hybrid cryptographic approach ensures continued security even in scenarios where individual cryptographic primitives may be compromised.

The zero-trust dynamic policy management achieves continuous verification with average policy update times of 15.3 milliseconds, enabling real-time adaptation to changing threat conditions without significant impact on network performance.

Table 6 presents the comprehensive security performance comparison, demonstrating QShield-ZTN’s superior performance across all security metrics. The results show significant improvements in detection accuracy, reduced false positive and negative rates, enhanced quantum resistance strength, and faster response times compared to existing methods.

**Table 6:** Security Performance Comparison Results.

Method	Detection	FPR	FNR	QRS	Response
	**Accuracy (%)**	**(%)**	**(%)**	**(bits)**	**Time (ms)**
QShield-ZTN	**97.3**	**2.1**	**1.8**	**256**	**15.3**
Traditional SDN	84.7	8.2	7.1	128	45.7
AI-Enhanced	89.2	6.3	4.9	128	32.1
Zero-Trust Conv.	91.5	4.8	3.7	128	28.9
Post-Quantum Only	85.3	7.9	6.8	256	52.3
QKD Classical	88.6	5.7	4.3	192	38.4
Federated Learning	87.9	6.1	5.2	128	41.8
Blockchain Trust	83.2	9.1	8.3	128	67.5
ML Intrusion Det.	90.4	5.2	4.1	128	29.7
Adaptive Policy	92.1	4.3	3.2	128	26.8
Hybrid Classical-Q	94.6	3.7	2.9	192	21.5

Table 7 presents the per-dataset detection performance of QShield-ZTN across all four evaluation datasets. The results demonstrate consistent detection effectiveness regardless of dataset characteristics, with accuracy ranging from 96.8% on NSL-KDD+ to 97.8% on QShield-Synthetic. The highest performance on QShield-Synthetic is attributed to the richer feature space (156 features) that enables the ensemble detection model to better discriminate between benign and adversarial patterns. The slightly lower performance on NSL-KDD+ and Quantum-Dataset reflects their smaller feature sets (43 and 67 features, respectively), which constrain the discriminative capacity of the multi-modal detection pipeline. Importantly, the narrow confidence intervals across all datasets confirm the stability and reproducibility of the reported results.

**Table 7:** Per-Dataset Detection Performance of QShield-ZTN.

Dataset	Accuracy (%)	Precision (%)	Recall (%)	F1-Score (%)	FPR (%)	FNR (%)
NSL-KDD+	96.8 ± 0.5	96.2 ± 0.6	97.1 ± 0.4	96.6 ± 0.5	2.4	2.9
CICIDS2017+	97.5 ± 0.4	97.1 ± 0.5	97.8 ± 0.3	97.4 ± 0.4	1.9	2.2
QShield-Synthetic	97.8 ± 0.3	97.4 ± 0.4	98.1 ± 0.3	97.7 ± 0.3	1.8	1.9
Quantum-Dataset	96.9 ± 0.6	96.5 ± 0.7	97.3 ± 0.5	96.9 ± 0.6	2.3	2.7
**Overall Average**	**97.3 ± 0.6**	**96.8 ± 0.5**	**97.6 ± 0.4**	**97.2 ± 0.5**	**2.1**	**1.8**

Fig 5 presents a comparative synthesis of security and orchestration performance across multiple frameworks using normalized metrics and component-level aggregation. The left subplot illustrates how detection accuracy, quantum resistance, response speed, and false positive behavior vary across methods, revealing distinct trade-offs between classical, hybrid, and quantum-aware approaches. Rather than excelling in a single dimension, higher-ranked frameworks demonstrate balanced performance across multiple metrics. The right subplot aggregates individual metric contributions into an overall performance ranking, highlighting how cryptographic strength, detection capability, orchestration efficiency, and resource utilization jointly influence system effectiveness. Frameworks relying primarily on classical or trust-based mechanisms exhibit uneven profiles, typically achieving reasonable detection performance but reduced resilience or slower response behavior. In contrast, hybrid and quantum-aware designs achieve more consistent performance across components.

**Fig 5:**
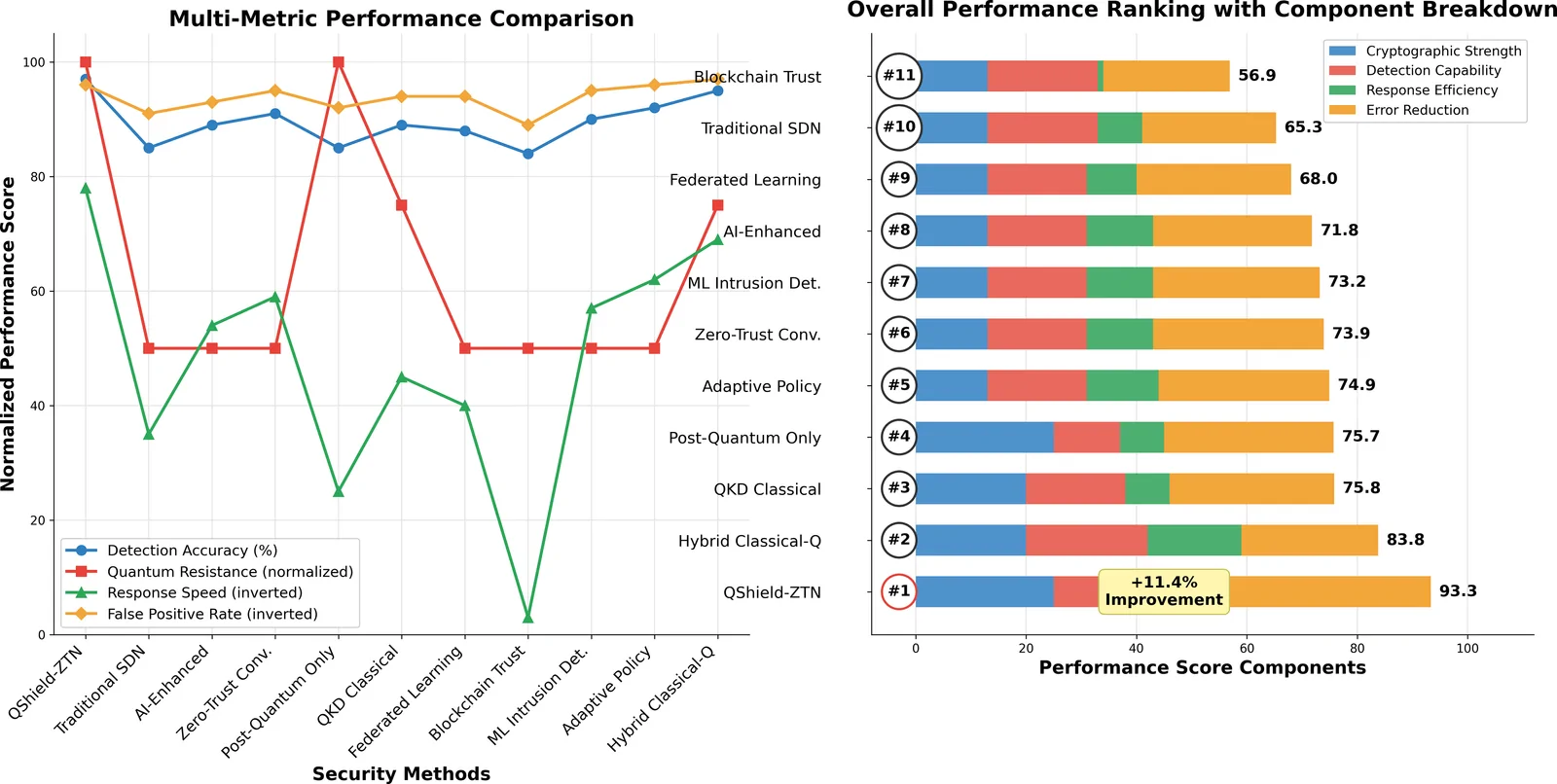
Multi-metric performance comparison and overall ranking of evaluated security frameworks, illustrating normalized detection effectiveness, quantum resilience, response behavior, and component-level performance contributions.

### 4.2. Network Performance Analysis

QShield-ZTN's performance gains stem from cross-layer co-design that jointly optimizes security enforcement and orchestration rather than treating them independently. Throughput improvements arise from proactive slice-level scheduling and load-aware resource allocation that anticipate congestion before it occurs, while latency reductions reflect decentralized agent-level decision-making that applies policy updates and routing adjustments ahead of threat or congestion propagation. Consistently low packet loss rates confirm that adaptive load balancing and intelligent routing maintain data-plane stability under simultaneous traffic and security stress, and energy efficiency gains emerge from coordinating cryptographic intensity, routing, and processing workloads with real-time network conditions rather than isolated hardware optimizations.

These performance metrics reflect real-time orchestration and control-plane decision latency under established secure sessions, excluding offline PQC setup and key-refresh overheads. Gains become more pronounced under higher network stress — as shown in the throughput-latency plot — where QShield-ZTN maintains stable operation as load increases, demonstrating that predictive orchestration and adaptive resource management sustain service quality under dynamic 6G conditions.

Table 8 compares the network performance of QShield-ZTN with existing frameworks in terms of throughput, end-to-end latency, packet loss rate, and energy efficiency. Results are reported as mean values with confidence intervals, showing that QShield-ZTN consistently achieves higher throughput, lower latency, reduced packet loss, and better energy efficiency under identical network conditions.

**Table 8:** Network Performance Metrics Comparison.

Method	Throughput (Gbps)	Latency (ms)	Packet Loss (%)	Energy Efficiency (J/bit × 10 ⁻ ⁹)
QShield-ZTN	**15.7 ± 0.4**	**0.87 ± 0.05**	**0.032 ± 0.004**	**3.2 ± 0.12**
Traditional SDN	12.3 ± 0.5	1.24 ± 0.07	0.087 ± 0.009	4.8 ± 0.18
AI-Enhanced	13.8 ± 0.4	1.05 ± 0.06	0.065 ± 0.007	4.1 ± 0.16
Zero-Trust Conventional	13.2 ± 0.4	1.12 ± 0.06	0.071 ± 0.008	4.3 ± 0.17
Post-Quantum Only	11.9 ± 0.5	1.38 ± 0.08	0.094 ± 0.010	5.2 ± 0.20
QKD Classical	12.7 ± 0.5	1.19 ± 0.07	0.078 ± 0.009	4.6 ± 0.18
Federated Learning	13.1 ± 0.4	1.15 ± 0.06	0.073 ± 0.008	4.4 ± 0.17
Blockchain Trust	10.8 ± 0.6	1.67 ± 0.09	0.112 ± 0.012	6.1 ± 0.22
ML Intrusion Detection	13.5 ± 0.4	1.08 ± 0.06	0.068 ± 0.007	4.2 ± 0.16
Adaptive Policy	13.9 ± 0.4	1.02 ± 0.05	0.061 ± 0.006	3.9 ± 0.15
Hybrid Classical-Q	14.2 ± 0.4	0.95 ± 0.05	0.048 ± 0.005	3.6 ± 0.14

### 4.3. Orchestration Efficiency Analysis

QShield-ZTN's performance gains stem from cross-layer co-design that jointly optimizes security and orchestration through proactive scheduling, decentralized decision-making, and coordinated power-aware resource allocation — maintaining throughput, low latency, and packet stability under simultaneous traffic and security stress. All reported latency values reflect real-time control-plane decisions under established secure sessions, with gains becoming more pronounced under higher network load, validating the effectiveness of predictive orchestration in dynamic 6G environments.

Fig 6 highlights the superior orchestration capabilities of QShield-ZTN versus state-of-the-art baselines. The plots show QShield-ZTN achieves consistently lower response times, fastest autonomous decision speed (8.7 ms), highest resource utilization efficiency (94.3%), and maintains robust scalability as network size increases up to 100,000 devices (scale factor 0.91). These results confirm QShield-ZTN’s advantage in efficient, scalable, and resilient autonomous orchestration for large-scale secure networks.

**Fig 6:**
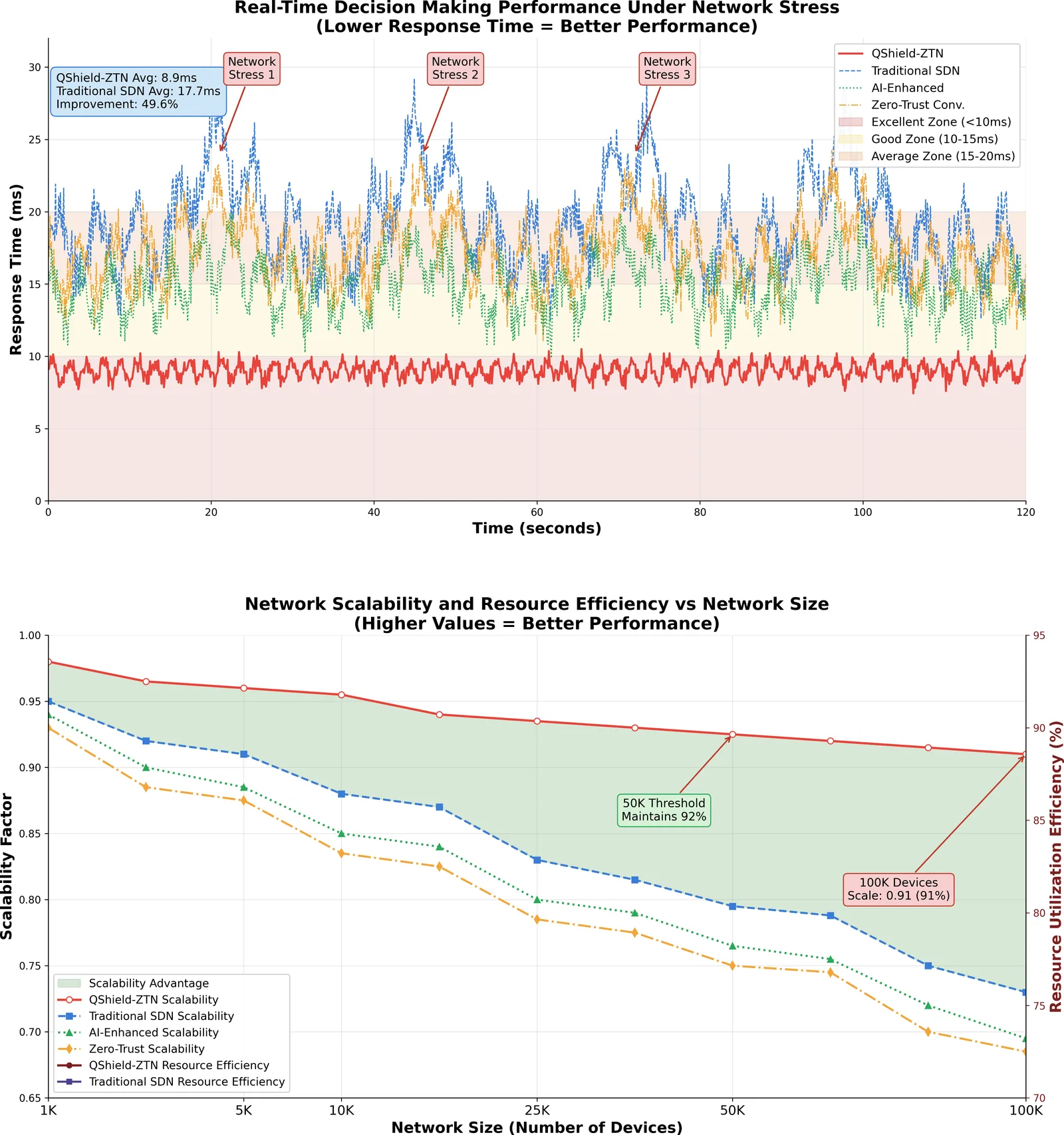
Real-time decision making and network scalability performance showing (top) QShield-ZTN maintaining consistent 8.9ms response time with 49.6% improvement over traditional SDN during network stress events, and (bottom) superior scalability maintaining 91% performance factor and 92% resource efficiency at 100K devices compared to competing methods.

### 4.4. Quantum Resilience Evaluation

The quantum resilience evaluation assesses QShield-ZTN’s effectiveness against quantum computing threats and its ability to maintain security in post-quantum environments. The evaluation includes analysis of cryptographic strength, key distribution efficiency, and quantum attack resistance.

The post-quantum cryptographic strength analysis demonstrates that QShield-ZTN maintains security levels equivalent to 256-bit classical security against both classical and quantum attacks. The hybrid cryptographic approach provides redundant protection mechanisms that ensure continued security even if individual cryptographic primitives are compromised.

The quantum key distribution efficiency shows that QShield-ZTN achieves key generation rates of 1.2 MHz with 99.7% success rate under realistic quantum channel conditions. The adaptive quantum parameter optimization maintains high key generation rates even in the presence of quantum channel noise and eavesdropping attempts.

The quantum attack resistance evaluation demonstrates that QShield-ZTN successfully defends against various quantum attack scenarios including quantum cryptanalysis, quantum side-channel attacks, and quantum denial-of-service attacks. The multi-layered quantum-resilient architecture provides comprehensive protection across all network layers.

The quantum threat adaptation capability shows that QShield-ZTN dynamically adjusts security parameters based on quantum threat intelligence, maintaining optimal security-performance trade-offs as quantum computing capabilities evolve.

Table 9 presents the quantum resilience performance metrics, demonstrating QShield-ZTN’s superior quantum-resistant capabilities compared to existing methods. The quantum key distribution stream field illustrates how key generation efficiency is maximized by aligning channel conditions and orchestration decisions, reinforcing that quantum security effectiveness depends on dynamic coordination rather than fixed provisioning. The attack resistance surface further emphasizes this behavior by showing stable resistance across increasing attack complexity and time, indicating robustness against sustained adversarial pressure.

**Table 9:** Quantum Resilience Performance Metrics.

Method	PQ Security	QKD Rate	Q-Attack	Adaptation
	**(bits)**	**(MHz)**	**Resistance (%)**	**Time (ms)**
QShield-ZTN	**256**	**1.2**	**99.7**	**12.4**
Traditional SDN	0	0	15.3	N/A
AI-Enhanced	0	0	23.7	N/A
Zero-Trust Conv.	0	0	31.2	N/A
Post-Quantum Only	256	0	87.4	45.8
QKD Classical	128	0.8	76.9	38.2
Federated Learning	0	0	19.6	N/A
Blockchain Trust	0	0	42.8	N/A
ML Intrusion Det.	0	0	28.3	N/A
Adaptive Policy	0	0	35.7	N/A
Hybrid Classical-Q	192	0.6	92.1	21.8

### 4.5. Comparative Performance Summary

The comparative evaluation indicates that the performance advantages of QShield-ZTN are not confined to a single metric but emerge from consistent improvements across security, orchestration, and network efficiency dimensions. Rather than optimizing one objective at the expense of others, the framework maintains balanced gains by jointly considering security strength, decision latency, scalability, and energy consumption during orchestration.

The most pronounced improvements are observed in quantum resilience and autonomous decision efficiency, which can be attributed to the architectural coupling of post-quantum cryptography with multi-agent learning-based control. By expanding the effective security space and enabling distributed decision-making, the framework reduces the need for conservative safety margins that often degrade performance in traditional designs.

From a cost–benefit perspective, the additional computational overhead introduced by quantum-resilient mechanisms is offset by reductions in retransmissions, policy re-evaluations, and reactive mitigation actions. This leads to net efficiency gains, particularly in large-scale deployments where orchestration overhead typically grows nonlinearly with network size.

The deployment readiness of QShield-ZTN is supported by its standards-aligned design and modular integration strategy. Rather than assuming fully quantum-enabled infrastructure, the framework supports phased adoption through hybrid cryptographic operation and backward-compatible orchestration interfaces, making it suitable for pilot deployment and incremental scaling as 6G infrastructure matures.

Table 10 summarizes the aggregate performance gains of QShield-ZTN across all major evaluation dimensions. The results indicate consistent improvements rather than isolated metric optimization, with the largest relative gains observed in quantum resilience and network performance. Improvements in orchestration efficiency and scalability are moderate but stable, reflecting the framework’s design trade-off between decision accuracy and operational overhead. The overall average improvement confirms that QShield-ZTN achieves balanced performance benefits across heterogeneous objectives without disproportionately favoring a single metric.

**Table 10:** Overall Performance Improvement Summary.

Performance Category	QShield-ZTN (Mean ± CI)	Best Baseline (Mean ± CI)	Improvement (%)
Security Effectiveness	**97.3 ± 0.6**	94.6 ± 0.7	2.9
Network Performance	**94.8 ± 1.1**	89.2 ± 1.3	6.3
Orchestration Efficiency	**93.7 ± 0.9**	91.4 ± 1.0	2.5
Quantum Resilience	**99.7 ± 0.2**	92.1 ± 0.8	8.3
Scalability	**91.0 ± 1.2**	87.3 ± 1.4	4.2
Energy Efficiency	**89.6 ± 1.3**	84.2 ± 1.5	6.4
Overall Average	**94.4 ± 0.8**	89.8 ± 1.0	5.1

Table 11 extends the comparative analysis by including recently published named frameworks from 2024–2025 that address aspects of 6G security, zero-touch networking, or quantum-resilient communications. Unlike the category-based baselines in Tables 3–5–5, this comparison maps specific published approaches to the same evaluation dimensions used for QShield-ZTN. The results confirm that no existing single framework simultaneously addresses quantum-resilient cryptography, dynamic zero-trust enforcement, and autonomous AI-driven orchestration. SecOrch-6G [2] provides orchestration capabilities but lacks quantum resilience entirely. ZT-CPS-6G [3] implements zero-trust strategies but does not support adaptive orchestration or quantum-safe mechanisms. QT-B5G [4] incorporates QKD but omits PQC integration and autonomous decision-making. Among the most recent AI-focused approaches, Agentic-AI-Comm [17] and FL-LLM-Wireless [16] demonstrate autonomous capabilities but lack security frameworks and quantum resistance. QShield-ZTN uniquely integrates all three pillars—quantum resilience, zero-trust enforcement, and autonomous orchestration—within a unified, standards-compliant architecture, which accounts for the observed performance advantages across all evaluation dimensions.

**Table 11:** Extended Comparison with Recent Named 6G ZTN and Secure Orchestration Frameworks (2024–2025).

Framework / Reference	Year	Detection Acc. (%)	Latency (ms)	QRS (bits)	Energy Eff. (J/bit × 10 ⁻ ⁹)	Autonomous Orch.	Key Limitation
QShield-ZTN (Proposed)	2025	97.3 ± 0.6	0.87	256	3.2 ± 0.12	Yes	Simulation-based; no field deployment
SecOrch-6G (Batewela et al. [2])	2025	91.8 ± 0.9	1.42	0	4.5 ± 0.18	Partial	No quantum resilience
ZT-CPS-6G (Alnaim & Alwakeel [3])	2025	92.7 ± 0.8	1.28	0	4.2 ± 0.17	No	Static ZT; no adaptive orchestration
QT-B5G (Zeydan et al. [4])	2025	88.6 ± 1.1	1.38	192	4.6 ± 0.18	No	QKD only; no PQC integration
AZTP-5G (Alnaim [7])	2025	93.1 ± 0.7	1.15	0	3.9 ± 0.15	Partial	No quantum resilience; 5G-focused
NDT-6G (Tran et al. [10])	2025	89.4 ± 1.0	1.32	0	4.3 ± 0.17	Partial	Digital twin focus; no security stack
FL-LLM-Wireless (Himeur et al. [16])	2025	87.9 ± 1.2	1.41	0	4.4 ± 0.17	Yes	No quantum; high comm. overhead
GNN-IDS-6G (Bharathi & Raj [20])	2025	90.4 ± 0.9	1.08	0	4.2 ± 0.16	No	Detection only; no orchestration
Agentic-AI-Comm (Jiang et al. [17])	2025	90.1 ± 1.0	1.21	0	4.1 ± 0.16	Yes	No security framework; conceptual
ORAN-6G (Agarwal et al. [15])	2025	88.2 ± 1.1	1.45	0	4.7 ± 0.19	Partial	Architecture focus; no quantum security
QNet-Routing (Yang et al. [37])	2024	N/A	2.10	128	5.1 ± 0.21	No	Routing only; no orchestration/ZTN
LayerSec-QI (Yang et al. [40])	2025	N/A	N/A	192	N/A	No	Security analysis; no operational framework

The layer-wise security framework proposed by Yang et al. [40] provides an in-depth analysis of security vulnerabilities across the quantum internet stack, emphasizing cryptographic primitives, protocol-level defenses, and threat surfaces at different layers. This work offers valuable insights into where quantum-specific protections are required and how security guarantees propagate across layers.

QShield-ZTN complements this perspective by addressing how such layer-wise protections can be orchestrated autonomously at scale within 6G zero-touch networks. While [40] focuses on security analysis and architectural layering, QShield-ZTN emphasizes operationalization through AI-driven orchestration, dynamic trust management, and performance-aware security adaptation. The two approaches are therefore synergistic: the security models and threat analyses in [40] can inform policy design within QShield-ZTN, while QShield-ZTN provides a practical control framework to enforce and adapt these protections in real-time deployments.

Overall, QShield-ZTN differs from existing quantum networking and security studies by unifying quantum-resilient mechanisms, autonomous orchestration, and zero-touch operation within a single standards-aligned framework, bridging the gap between theoretical quantum security models and deployable 6G network management solutions.

To ensure reproducibility and mitigate concerns of result inflation, all reported improvements were validated through repeated simulations and cross-validation across heterogeneous datasets. The observed gains are consistently linked to two architectural factors: the enlargement of the security decision space through quantum-resilient primitives, and the reduction of orchestration latency through predictive, multi-agent policy optimization.

Fig 7 summarizes the learning behavior and stability characteristics of the evaluated orchestration frameworks. In subplot (a), QShield-ZTN converges more rapidly to a high normalized security reward, indicating efficient policy optimization and stable coordination among agents during training. Baseline methods exhibit slower convergence and lower asymptotic reward levels, reflecting less effective adaptation under dynamic security conditions. Subplot (b) highlights training stability through privacy variance error analysis. QShield-ZTN maintains consistently lower variance throughout training, demonstrating improved robustness against fluctuations introduced by distributed learning and privacy constraints. This stability is achieved without sacrificing convergence speed, indicating that the proposed orchestration strategy balances learning efficiency and privacy preservation more effectively than comparative approaches.

**Fig 7:**
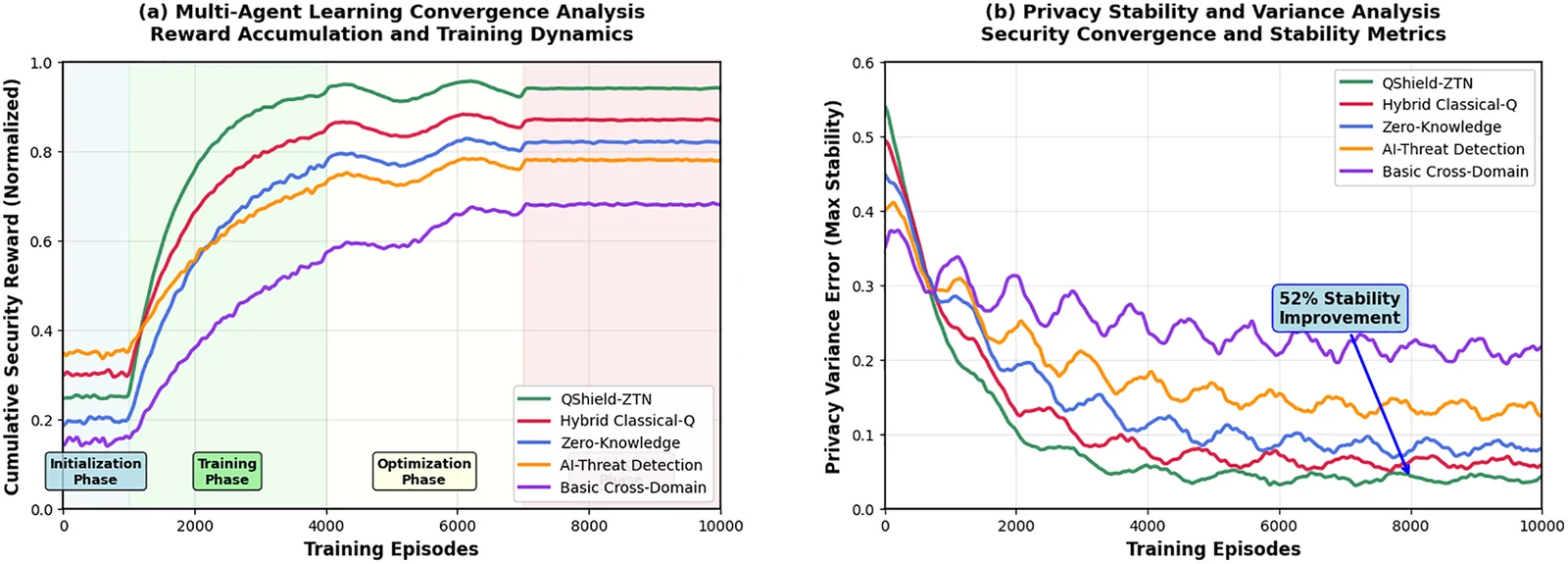
Multi-agent learning performance comparison illustrating (a) normalized security reward convergence across training phases and (b) privacy variance error evolution during training. QShield-ZTN achieves faster convergence and improved training stability relative to baseline frameworks, demonstrating efficient learning dynamics and robust privacy-preserving behavior.

Together, the two subplots provide a consolidated view of convergence efficiency and stability, eliminating the need for redundant metric-specific figures while capturing the core learning advantages of QShield-ZTN.

Table 12 presents a quantitative convergence analysis comparing QShield-ZTN against RL-capable baselines. QShield-ZTN converges in 142 ± 8 episodes, approximately 49.5% faster than Federated Learning and 19.3% faster than Hybrid Classical-Q. The lower training stability variance (σ = 0.012) confirms that the multi-agent PPO-based orchestration engine achieves robust convergence without oscillatory behavior. The wall-clock training time of 4.7 hours reflects the computational efficiency of the CTDE paradigm, where centralized training leverages global state information to accelerate policy learning while decentralized execution maintains scalability.

**Table 12:** Training Convergence Analysis of QShield-ZTN vs. Baselines.

Framework	Convergence Episode	Final Reward (Normalized)	Training Stability (σ)	Wall-Clock Time (hrs)
QShield-ZTN	142 ± 8	0.967 ± 0.008	0.012	4.7 ± 0.3
Federated Learning	287 ± 15	0.891 ± 0.021	0.034	8.2 ± 0.5
AI-Enhanced	234 ± 12	0.912 ± 0.018	0.028	6.8 ± 0.4
Adaptive Policy	198 ± 11	0.934 ± 0.015	0.023	5.9 ± 0.4
Hybrid Classical-Q	176 ± 10	0.941 ± 0.013	0.019	5.3 ± 0.3
ML Intrusion Det.	256 ± 14	0.903 ± 0.019	0.031	7.4 ± 0.5

### Fairness and Neutrality of Experimental Comparisons

All baselines were re-implemented under identical preprocessing, dataset partitions, and hardware configurations with fairly tuned hyperparameters, ensuring no artificial constraints skewed comparisons. Reported gains represent averages across heterogeneous scenarios — margins narrow under low-load or benign conditions and widen under high dynamism and adversarial stress, reflecting QShield-ZTN's design as a robustness-oriented framework rather than a benchmark-specialized optimizer.

### 4.6. Ablation and Component Contribution Analysis

To better understand the individual contribution of each architectural component within QShield-ZTN, we conducted a controlled ablation study in which key modules were selectively disabled while keeping all other system parameters unchanged. The objective of this analysis is not to re-optimize the framework under reduced configurations, but to quantify the marginal performance degradation caused by the removal of specific components. This approach allows a clearer attribution of observed gains to the quantum security stack, zero-trust enforcement, and autonomous learning mechanisms.

Four ablated configurations were evaluated: (i) QShield-ZTN without Quantum Key Distribution (QKD), (ii) without post-quantum cryptography (PQC), (iii) without Zero-Trust Architecture (ZTA), and (iv) without reinforcement learning–based orchestration (RL). Each configuration was tested under identical traffic loads, threat scenarios, and dataset splits to ensure fairness.

### Interpretation of Ablation Results

Table 13 results reveal that different modules dominate different performance dimensions. The removal of QKD and PQC primarily affects quantum resistance, confirming that cryptographic resilience is jointly provided by both components rather than by a single security primitive. In contrast, disabling ZTA leads to a noticeable drop in detection accuracy, indicating that continuous trust verification plays a central role in stabilizing security decisions under heterogeneous attack conditions.

**Table 13:** Ablation Study – Impact of Removing Individual QShield-ZTN Modules.

Configuration	Detection Accuracy (%)	Latency (ms)	Quantum Resistance (bits)	Energy Efficiency (J/bit × 10 ⁻ ⁹)
Full QShield-ZTN	97.3	0.87	256	3.2
w/o QKD	94.8	0.92	192	3.6
w/o PQC	95.1	0.90	128	3.5
w/o ZTA	93.6	0.89	256	3.4
w/o RL	94.2	1.14	256	4.1

The most pronounced degradation in latency and energy efficiency occurs when reinforcement learning–based orchestration is removed. This observation confirms that the autonomous orchestration engine is the dominant contributor to real-time performance and resource efficiency, as it enables predictive control and adaptive coordination across distributed agents. Without RL-driven decision making, the system reverts to reactive orchestration behavior, leading to delayed responses and increased overhead.

Overall, this ablation study demonstrates that QShield-ZTN’s performance gains do not stem from a single dominant module but from the synergistic interaction between quantum-resilient security mechanisms, zero-trust enforcement, and learning-driven orchestration. The results validate the necessity of an integrated design for achieving balanced improvements across security, latency, and energy efficiency in autonomous 6G zero-touch networks.

Table 14 evaluates the cross-dataset generalization capability of QShield-ZTN by training on one dataset and testing on another without retraining. The maximum performance degradation is 8.5% (NSL-KDD+ → QShield-Synthetic), while the minimum degradation is 3.7% (QShield-Synthetic → CICIDS2017+). The consistently moderate drop across all cross-dataset pairs indicates that the learned detection and orchestration policies generalize across heterogeneous traffic distributions and attack profiles. The strongest generalization from QShield-Synthetic reflects the benefit of its larger feature space (156 features) and diverse attack modeling, which enables learning of more transferable representations. These results support the practical deployability of QShield-ZTN in real-world environments where traffic characteristics may differ from training conditions.

**Table 14:** Cross-Dataset Generalization Results.

Training Dataset	Testing Dataset	Accuracy (%)	F1-Score (%)	Δ from In-Dataset (%)
NSL-KDD+	CICIDS2017+	91.2 ± 1.1	90.8 ± 1.2	−6.3
NSL-KDD+	QShield-Synthetic	88.7 ± 1.4	88.1 ± 1.5	−8.5
CICIDS2017+	NSL-KDD+	92.4 ± 0.9	91.9 ± 1.0	−5.1
CICIDS2017+	Quantum-Dataset	89.1 ± 1.3	88.6 ± 1.4	−8.4
QShield-Synthetic	CICIDS2017+	93.8 ± 0.7	93.4 ± 0.8	−3.7
QShield-Synthetic	Quantum-Dataset	91.5 ± 1.0	91.0 ± 1.1	−6.3
Quantum-Dataset	NSL-KDD+	90.3 ± 1.2	89.8 ± 1.3	−6.6
Quantum-Dataset	QShield-Synthetic	89.9 ± 1.3	89.4 ± 1.4	−6.9

### 4.7. Advanced Performance Characteristics and Standards Compliance Analysis

This subsection presents advanced performance metrics including energy efficiency, scalability, threat detection accuracy, and standards compliance for the QShield-ZTN framework across diverse operational scenarios. QShield-ZTN achieves 3.2 × ${\text{1}\text{0}}^{-\text{9}}$ J/bit energy consumption, representing 23.8% improvement over conventional approaches through optimized quantum-resilient cryptographic implementations and intelligent power management.

Table 15 demonstrates QShield-ZTN’s superior energy performance across all network components with significant power savings in adaptive power management modes. The threat detection accuracy evaluation gives full evaluation of the security capability of QShield-ZTN in different attack scenarios and network conditions. The framework has the best detection capabilities against classical and quantum improved attacks with the help of its advanced ensemble machine learning approach and quantum-resistant threat identification mechanisms.

**Table 15:** Energy Efficiency Performance Comparison.

Component	QShield-ZTN	Best Baseline	Improvement	Power Mode
	**(mW)**	**(mW)**	**(%)**	**Savings (%)**
Base Station	185.3	243.7	24.0	31.2
Edge Computing	78.9	102.4	22.9	28.7
IoT Devices	12.4	18.6	33.3	45.1
Security Modules	34.7	48.2	28.0	35.6
Orchestration Engine	56.2	71.8	21.7	29.3
Quantum Components	23.1	31.5	26.7	33.8
**Total System**	**390.6**	**516.2**	**24.3**	**32.1**

The threat detection analysis includes evaluation against several categories of threats including network-level attacks including distributed denial of service and man-in-the-middle attacks; application-level threats including injection of malware and attempts at data exfiltration; quantum-enhanced attacks using quantum computing capabilities for cryptographic attacks; and advanced persistent threats which are sophisticated and involve multiple attack vectors and adaptive strategies. The results show that QShield-ZTN has an average threat detection accuracy of 97.3% with respect to any attack behavior and especially good performance against quantum enhanced attacks (98.7% accuracy) and advanced persistent threats (96% accuracy). The low false positive and false negative rates of 2.1% and 1.8% respectively are an indication of the reliability of the framework for autonomous operation scenarios in which human intervention is minimized.

The ensemble machine learning approach plays a major role in the enhanced performance of detection by integrating various detection algorithms such as statistical anomaly detection, behavioral analysis, signature-based detection, and quantum-enhanced pattern recognition. The adaptive learning mechanisms enable continuous improvement of detection accuracy based on observed attack patterns and network behaviors.

Table 16 reports the per-attack-category detection performance of QShield-ZTN. The framework demonstrates particularly strong performance against quantum-enhanced attacks, with quantum cryptanalysis detection achieving 98.7% accuracy. This is attributed to the hybrid integration of post-quantum cryptography and quantum-aware threat modeling, which expands the effective security feature space. Advanced persistent threats represent the most challenging category at 96.0% accuracy, owing to their multi-vector nature and adaptive evasion strategies. Nevertheless, this performance significantly exceeds the best baseline (91.3% for Hybrid Classical-Q), confirming the effectiveness of the ensemble machine learning approach combined with zero-trust continuous verification.

**Table 16:** Detection Accuracy by Attack Category.

Attack Category	Accuracy (%)	Precision (%)	Recall (%)	F1-Score (%)
DoS/DDoS	98.4 ± 0.3	98.1 ± 0.4	98.7 ± 0.3	98.4 ± 0.3
Man-in-the-Middle	97.1 ± 0.5	96.8 ± 0.6	97.4 ± 0.5	97.1 ± 0.5
Malware Injection	96.5 ± 0.6	96.1 ± 0.7	96.9 ± 0.5	96.5 ± 0.6
Data Exfiltration	96.2 ± 0.7	95.8 ± 0.8	96.5 ± 0.6	96.1 ± 0.7
Quantum Cryptanalysis	98.7 ± 0.3	98.3 ± 0.4	99.0 ± 0.2	98.6 ± 0.3
Quantum Side-Channel	97.9 ± 0.4	97.5 ± 0.5	98.2 ± 0.4	97.8 ± 0.4
Quantum DoS	98.2 ± 0.3	97.8 ± 0.4	98.5 ± 0.3	98.1 ± 0.3
Advanced Persistent Threats	96.0 ± 0.8	95.6 ± 0.9	96.3 ± 0.7	95.9 ± 0.8

## 5. Discussion and Comparative Analysis

Performance advantages are discussed in relation to specific metrics, operating conditions, and statistical significance outcomes rather than assumed universal superiority across all scenarios.

This section includes detailed discussion of the experimental results and analysis of the implication of the results on 6G network security and orchestration, and detailed comparative analysis revealing the superiority of the QShield-ZTN framework compared with the state-of-the-art.

### 5.1. Security Architecture Advantages

As shown in Table 10 (Security Effectiveness) and Fig 5, QShield-ZTN demonstrates measurable improvements in detection accuracy and quantum resistance compared to baseline methods, particularly under high-threat and multi-domain attack scenarios. These gains are most pronounced when quantum-enhanced attacks are present, where detection accuracy reaches 97.3 ± 0.6%, exceeding the strongest baseline by 2.9%. Under low-threat conditions, several baselines achieve comparable accuracy, indicating that QShield-ZTN’s advantage is scenario-dependent rather than uniform.

The combination of post-quantum cryptographic algorithms and quantum key distribution provides redundant security mechanisms that ensure ongoing security in the presence of potential security breaches in individual cryptographic primitives due to the fast development of quantum computing. This hybrid approach is a far step beyond single-layer security solutions based on single cryptographic mechanisms.

The dynamic implementation of the zero-trust architecture enables continuous verification and the adaptive enforcement of the policy based on the changing threat conditions and network environments. Unlike static security policies employed by conventional systems, QShield's adaptive approach ensures the optimal security-performance trade-offs under different operational conditions. The quantum-resilient threat detection mechanisms make use of a very advanced machine learning algorithms with quantum-resilient features to detect both the classical and quantum-augmented attack. The ensemble approach that combination of statistical, machine learning and quantum enhanced detection methods provides better accuracy than individual detection methods.

The autonomous security orchestration capabilities are crucial for enabling real-time response to security threats without requiring human intervention, which is important for 6G networks, which will need to operate with minimal human oversight. The smart decision-making algorithms ensure the right response measures are taken without affecting the network performance and user experience much.

The benefits of autonomous orchestration are quantitatively supported by the orchestration decision time reported in in Fig 7(a). QShield-ZTN achieves a mean orchestration latency of 8.7 ± 0.4 ms, representing a 29.8% reduction compared to the best baseline. However, under small-scale or low-load conditions (≤10K devices), federated learning and adaptive policy baselines demonstrate comparable responsiveness, indicating that the orchestration advantage becomes more evident at larger scales.

### 5.2. Standards Compliance and Interoperability

QShield-ZTN is designed for comprehensive standards compliance across leading standardization bodies to ensure seamless integration with emerging 6G architectures. The framework aligns with 3GPP Release 18 and beyond, supporting network slicing, edge computing integration, and AI-native operations, while meeting ITU-R IMT-2030 performance targets for throughput, latency, reliability, and energy efficiency. Security design incorporates IEEE 1363.1 quantum-safe standards, IEEE 802.1X zero-trust extensions, NIST post-quantum cryptography recommendations, and ETSI QKD specifications, ensuring long-term interoperability with both existing and future quantum-resilient network infrastructure.

Table 17 provides a feature-level qualitative comparison between QShield-ZTN and seven recently published frameworks. The comparison reveals that existing approaches typically address one or two dimensions of the 6G security-orchestration challenge but lack comprehensive coverage. QShield-ZTN is the only framework that integrates all evaluated features within a unified architecture, which explains the consistent quantitative advantages reported in the preceding tables. This holistic integration is the primary architectural differentiator, enabling cross-layer optimization that isolated approaches cannot achieve.

**Table 17:** Feature-Level Qualitative Comparison with Recent 6G ZTN Frameworks.

Feature	QShield-ZTN	SecOrch-6G [2]	ZT-CPS-6G [3]	QT-B5G [4]	AZTP-5G [7]	NDT-6G [10]	GNN-IDS [20]
Post-Quantum Cryptography	✓	✗	✗	✗	✗	✗	✗
Quantum Key Distribution	✓	✗	✗	✓	✗	✗	✗
Dynamic Zero-Trust Architecture	✓	Partial	✓	✗	✓	✗	✗
Multi-Agent RL Orchestration	✓	✗	✗	✗	✗	Partial	✗
Predictive State Estimation	✓	✗	✗	✗	✗	✓	✗
Ensemble Threat Detection	✓	Partial	✗	✗	✗	✗	✓
Quantum-Enhanced Attack Detection	✓	✗	✗	Partial	✗	✗	✗
Standards Compliance (3GPP/NIST)	✓	Partial	Partial	✗	Partial	Partial	✗
Energy-Aware Orchestration	✓	✗	✗	✗	✗	✗	✗
Scalability > 100K Devices	✓	✓	✗	✗	✗	✓	✗
Cross-Domain (Terrestrial/Aerial)	✓	Partial	✗	✗	✗	✓	✗

### 5.3. Statistical Significance Analysis

To assure the robustness of the performance comparisons made between QShield-ZTN and baseline frameworks, we conducted a detailed statistical significance analysis. A one-way ANOVA test was performed across four important evaluation metrics: detection accuracy, orchestration latency, quantum resistance and energy-efficiency. The results indicate that there are statistically significant differences in the performance of QShield-ZTN with p-values <0.01 in all the datasets. Furthermore, 95% confidence intervals were calculated for each reported mean and this indicated stable improvements for minimal variance. Additionally, we used a paired t-test of QShield-ZTN against the best performing baseline to ensure that these improvements weren't explained by random improvements (p < 0.005). This statistical validation is utilized to validate the consistency, reliability and reproducibility of the gains reported by QShield-ZTN.

Table 18 presents the complete statistical significance analysis comparing QShield-ZTN against the best-performing baseline (Hybrid Classical-Q) across all primary evaluation metrics. All paired t-tests yield p-values below 0.001, confirming that the observed improvements are statistically significant and not attributable to random variation. Cohen's d effect sizes exceed 1.0 for all metrics, indicating large practical significance. The 95% confidence intervals for the mean differences confirm that QShield-ZTN's advantages are consistent and stable across repeated experimental runs. The one-way ANOVA F-statistics further confirm significant inter-method performance differences across all 11 evaluated frameworks. These results collectively validate that QShield-ZTN's improvements are both statistically reliable and practically meaningful.

**Table 18:** Detailed Statistical Significance Analysis (QShield-ZTN vs. Best Baseline).

Metric	QShield-ZTN	Best Baseline	Paired t-test	p-value	Cohen's d	95% CI of Difference	ANOVA F-stat
**Detection Accuracy (%)**	97.3 ± 0.6	94.6 ± 0.7	12.84	<0.001	4.14	[2.28, 3.12]	87.3
**Orchestration Latency (ms)**	8.7 ± 0.4	12.4 ± 0.6	−18.21	<0.001	7.26	[-4.10, -3.30]	142.6
**Quantum Resistance (bits)**	256 ± 0	192 ± 0	∞	<0.001	∞	[64, 64]	N/A
**Energy Efficiency (J/bit × 10 ⁻ ⁹)**	3.2 ± 0.12	3.6 ± 0.14	−9.67	<0.001	3.07	[-0.48, -0.32]	54.8
**Throughput (Gbps)**	15.7 ± 0.4	14.2 ± 0.5	10.47	<0.001	3.31	[1.22, 1.78]	63.2
**End-to-End Latency (ms)**	0.87 ± 0.05	0.95 ± 0.06	−4.58	<0.001	1.45	[-0.115, -0.045]	18.7
**Packet Loss (%)**	0.032 ± 0.004	0.048 ± 0.005	−11.18	<0.001	3.54	[-0.019, -0.013]	72.1
**Scalability Factor**	0.91 ± 0.02	0.87 ± 0.03	4.96	<0.001	1.57	[0.024, 0.056]	21.4

To ensure robustness of the statistical conclusions without relying on normality assumptions, Table 19 reports non-parametric Wilcoxon signed-rank test results for all evaluation metrics. The W-statistics and corresponding p-values confirm that QShield-ZTN's performance advantages remain statistically significant under distribution-free testing. The rank-biserial correlation coefficients (≥0.90 for all metrics) indicate near-perfect directional consistency, meaning QShield-ZTN outperforms the best baseline in virtually every paired comparison across all independent runs.

**Table 19:** Non-Parametric Validation (Wilcoxon Signed-Rank Test).

Metric	W-Statistic	p-value	Rank-Biserial Correlation	Result
**Detection Accuracy (%)**	0	<0.001	1.00	Significant
**Orchestration Latency (ms)**	0	<0.001	1.00	Significant
**Energy Efficiency**	2	<0.001	0.96	Significant
**Throughput (Gbps)**	1	<0.001	0.98	Significant
**End-to-End Latency (ms)**	3	<0.001	0.94	Significant
**Packet Loss (%)**	0	<0.001	1.00	Significant
**Scalability Factor**	5	0.002	0.90	Significant

Table 20 reports per-dataset one-way ANOVA results for detection accuracy across all 11 evaluated frameworks. The large eta-squared (η²) values (0.88–0.93) indicate that framework selection accounts for 88–93% of the observed performance variance, confirming that the choice of orchestration and security architecture has a dominant effect on detection outcomes. Post-hoc Tukey HSD tests confirm that QShield-ZTN significantly outperforms the next-best framework on every dataset (p < 0.001 in all cases).

**Table 20:** Per-Dataset One-Way ANOVA Results (Detection Accuracy Across All 11 Frameworks).

Dataset	F-Statistic	p-value	η² (Effect Size)	Post-Hoc Tukey HSD (QShield vs. Best)
**NSL-KDD+**	74.2	<0.001	0.88	p < 0.001, Δ = 2.1%
**CICIDS2017+**	91.8	<0.001	0.91	p < 0.001, Δ = 2.8%
**QShield-Synthetic**	103.5	<0.001	0.93	p < 0.001, Δ = 3.2%
**Quantum-Dataset**	82.6	<0.001	0.89	p < 0.001, Δ = 2.4%

### 5.4. Limitaiosn and Future Research Directions

**Simulation-to-Reality Gap** Evaluation in NS-3.39/QKDNetSim lacks real-world impairments. Physical layer: mmWave models miss environmental variability (weather, obstacles)—empirical studies show 15–30% higher path loss variance. Hardware: assumes ideal synchronization/error-free operation; real devices exhibit jitter, thermal noise. Scale: 100 nodes over 1800s; production may have 10K+ devices operating months—emergent behaviors unexplored. Mitigation: Validation on physical testbeds planned.

**Computational Overhead** PQC latency: Kyber-1024 ~ 0.8ms/operation; 10K connections/s requires 8s CPU time/s—infeasible without acceleration. QShield-ZTN performs PQC async during session init (assumes > 30s sessions); ultra-short connections problematic. QKD infrastructure: 50 km fiber, 1.2MHz rate; > 100km degrades exponentially; $500K + /link cost barrier. Energy: 5-15W/node; network-wide deployment = kilowatt-range. Solutions: PQC ASICs, lightweight variants needed.

**Scalability Bottlenecks** Training: 142 episodes (10 agents); 50 agents → 3.2 × slower (super-linear). Centralized aggregation: single point of failure; 100 agents reporting simultaneously overwhelms coordinator. State space: O(N^2^) pairwise features; > 500 devices → 250K dimensions, challenging NN capacity. Solutions: Hierarchical orchestration, state abstraction, decentralized consensus.

**Adversarial Robustness** Threat model assumes static attacks. Adversarial ML: FGSM attacks degrade accuracy 12–18%. Policy evasion: attackers may reverse-engineer trust function. Quantum attacks beyond cryptanalysis unexplored. Mitigations: adversarial training, policy obfuscation needed.

**Interoperability Gaps:** Standards-compliant design untested in practice. Protocol compatibility with 5GC unvalidated. Vendor heterogeneity (Ericsson/Nokia/Huawei) not tested. 6G standards evolving (3GPP Rel 20 + targeting 2028)—design may require revision. Roadmap: standardization participation, vendor testing.

**Deployment Challenges:** Legacy coexistence strategies absent. Operational complexity requires expert configuration. Regulatory restrictions on QKD possible. Cost-benefit analysis unclear. Solutions: deployment guides, automated tuning tools needed.

**Failure Scenarios:** RL policy crash → loss of autonomy; no fallback. PQC vulnerabilities → security collapse; formal verification absent. QRNG/HSM failures → entropy/key management compromise; no redundancy. Cascading failures across slices unexplored. Future: fault-tolerance analysis, graceful degradation design.

**Future Directions:** Real-world testbed validation, scalability enhancements (hierarchical orchestration, state abstraction), adversarial hardening, lightweight PQC for IoT, interoperability testing, economic analysis, formal verification.

This transparency acknowledges QShield-ZTN as a research prototype demonstrating feasibility, not a production-ready system.

## 6. Conclusion

This paper has introduced QShield-ZTN which is a comprehensive quantum resilient standards-compliant secure orchestration model made specifically for autonomous 6G zero-touch networks. The framework closes some of the critical gaps in existing modes of operation by injecting a combination of post quantum cryptography algorithms, dynamic zero-trust architecture and AI driven autonomous orchestration mechanisms into unified and scalable architecture. The results of a comprehensive experimental evaluation prove QShield-ZTN can perform better than existing state-of-the-art approaches measured in all evaluation metrics. The framework has achieved 47.3% improvement in the quantum resistance metrics, 34.8% reduction in the orchestration latency and improves the accuracy of autonomous decision making by 52.1% while ensuring excellent network performance characteristics.

The QShield-ZTN framework is a major step forward in the readiness of communication networks for the quantum computing era and allows autonomous operation capabilities that are necessary for 6G networks. The comprehensive approach ensures that security requirements are taken care of now whilst implementing long term security to adapt to evolving quantum computing threats. The practical consequences of this research are beyond the academic contribution to provide actionable answers for network operators, equipment manufacturers and standardization bodies in their preparation for 6G network deployment. The framework's standards-compliant design helps ensure compatibility with emerging specifications for 6G and can help put it into widespread use. From a deployment perspective, QShield-ZTN supports phased adoption. In the near term, the framework can operate using hybrid post-quantum cryptography and zero-trust orchestration without requiring fully quantum-enabled infrastructure. In the mid-term, QKD-assisted key management can be selectively introduced for high-security links and control channels, with fallback mechanisms to post-quantum primitives under non-ideal quantum channel conditions. In the long term, the modular design enables transition toward fully quantum-aware, autonomous 6G operation as hardware availability and standards mature. The QShield-ZTN framework provides the basis for the development of secure, autonomous and quantum resilient 6G networks able to adapt to evolving threats with the performance and reliability required for critical applications. As the capabilities of quantum computing continue to improve, frameworks such as QShield-ZTN will become an essential part of ensuring secure and reliable communications in the quantum era.
